# Non-redundant and Redundant Roles of Cytomegalovirus gH/gL Complexes in Host Organ Entry and Intra-tissue Spread

**DOI:** 10.1371/journal.ppat.1004640

**Published:** 2015-02-06

**Authors:** Niels A. W. Lemmermann, Astrid Krmpotic, Jürgen Podlech, Ilija Brizic, Adrian Prager, Heiko Adler, Astrid Karbach, Yiquan Wu, Stipan Jonjic, Matthias J. Reddehase, Barbara Adler

**Affiliations:** 1 Institute for Virology and Research Center for Immunotherapy (FZI), University Medical Center of the Johannes Gutenberg-University Mainz, Mainz, Germany; 2 School of Medicine, University of Rijeka, Rijeka, Croatia; 3 Max von Pettenkofer-Institute for Virology, Ludwig-Maximilians-University Munich, Munich, Germany; 4 Research Unit Gene Vectors, Helmholtz Zentrum München—German Research Center for Environmental Health (GmbH), Munich, Germany; 5 Institute for Clinical and Molecular Virology, Friedrich-Alexander University Erlangen-Nürnberg, Erlangen, Germany; University of Wisconsin-Madison, UNITED STATES

## Abstract

Herpesviruses form different gH/gL virion envelope glycoprotein complexes that serve as entry complexes for mediating viral cell-type tropism *in vitro*; their roles *in vivo*, however, remained speculative and can be addressed experimentally only in animal models. For murine cytomegalovirus two alternative gH/gL complexes, gH/gL/gO and gH/gL/MCK-2, have been identified. A limitation of studies on viral tropism *in vivo* has been the difficulty in distinguishing between infection initiation by viral entry into first-hit target cells and subsequent cell-to-cell spread within tissues. As a new strategy to dissect these two events, we used a gO-transcomplemented ΔgO mutant for providing the gH/gL/gO complex selectively for the initial entry step, while progeny virions lack gO in subsequent rounds of infection. Whereas gH/gL/gO proved to be critical for establishing infection by efficient entry into diverse cell types, including liver macrophages, endothelial cells, and hepatocytes, it was dispensable for intra-tissue spread. Notably, the salivary glands, the source of virus for host-to-host transmission, represent an exception in that entry into virus-producing cells did not strictly depend on either the gH/gL/gO or the gH/gL/MCK-2 complex. Only if both complexes were absent in gO and MCK-2 double-knockout virus, *in vivo* infection was abolished at all sites.

## Author Summary

The role of viral glycoprotein entry complexes in viral tropism *in vivo* is a question central to understanding virus pathogenesis and transmission for any virus. Studies were limited by the difficulty in distinguishing between viral entry into first-hit target cells and subsequent cell-to-cell spread within tissues. Employing the murine cytomegalovirus entry complex gH/gL/gO as a paradigm for a generally applicable strategy to dissect these two events experimentally, we used a gO-transcomplemented ΔgO mutant for providing the complex exclusively for the initial cell entry step. In immunocompromised mice as a model for recipients of hematopoietic cell transplantation, our studies revealed an irreplaceable role for gH/gL/gO in initiating infection in host organs relevant to pathogenesis, whereas subsequent spread within tissues and infection of the salivary glands, the site relevant to virus host-to-host transmission, are double-secured by the entry complexes gH/gL/gO and gH/gL/MCK-2. As an important consequence, interventional strategies targeting only gO might be efficient in preventing organ manifestations after a primary viremia, whereas both gH/gL complexes need to be targeted for preventing intra-tissue spread of virus reactivated from latency within tissues as well as for preventing the salivary gland route of host-to-host transmission.

## Introduction

Herpesvirus entry is a complex process accomplished by a set of envelope glycoproteins that promote attachment of virus particles to host cells, recognition of host cell entry receptors, and fusion of the viral envelope with cellular membranes. All herpesviruses use a conserved core protein machinery consisting of glycoprotein gB and the glycoprotein complex gH/gL to promote the fusion process [1–2]. Recognition and binding to entry receptors on host cells *in vitro* may either be accomplished by the gH/gL core complex alone, by cooperation with other glycoproteins in the viral envelope, or by forming gH/gL complexes tightly binding additional viral proteins. Such multimeric gH/gL complexes are formed during virion assembly [1].

For Epstein-Barr virus (EBV), human herpesvirus 6, and human cytomegalovirus (HCMV) alternative multimeric gH/gL complexes that promote entry into distinct host cells have been identified [3–5]. During HCMV infection, two multimeric gH/gL complexes are formed: a pentameric gH/gL/pUL(128,130,131A) complex promoting entry into epithelial, endothelial, dendritic, and monocytic cells [6–11], and a trimeric gH/gL/gO complex promoting entry predominantly into fibroblasts ([12]; reviewed in [5]). Virus particles released from gO knock-out (ko) mutants are highly impaired on all cell types tested, whereas cell-associated focal virus spread in cell culture is not affected [13–14].

For EBV and HCMV it has been shown that host cells differentially route virus infection by influencing the gH/gL complex outfit of their virus progeny. In the case of EBV infection, replication in epithelial cells leads to production of virions rich in gH/gL/gp42 complexes targeting B cells, whereas replication in B cells mainly leads to incorporation of gp42-negative complexes into virions and thus to a virus progeny that targets epithelial cells [15]. Hence, replication in either B cells or epithelial cells induces a switch in cell type tropism. HCMV-infected cells have been shown to produce virus progeny heterogeneous in the amounts of the two gH/gL complexes and consequently in their cell type tropism. HCMV-infected fibroblasts release viruses that contain high or low amounts of gH/gL/pUL(128,130,131A) and are endotheliotropic or non-endotheliotropic, respectively [16]. Endothelial cells (EC), in contrast, release only virions that contain low amounts of gH/gL/pUL(128,130,131A) and retain those with a high gH/gL/pUL(128,130,131A) content, which renders spread of the latter cell-associated. Although host cells targeted by specific gH/gL complexes have been identified *in vitro*, it is not at all understood how alternative gH/gL complexes contribute to the infection *in vivo*. Clarification of the roles gH/gL complexes play *in vivo* will not only provide new insights into virus spread and host cell targeting, but will help to understand the roles of specific host cells in virus infection.

Infection of mice with murine cytomegalovirus (mCMV) is an accepted animal model for a CMV infection in its natural host and has revealed many general principles of CMV-host interaction. We have previously characterized the gH/gL/gO complex of mCMV, which *in vitro* is functionally homologous to the gH/gL/gO complex of HCMV [17]. Specifically, when mCMV gO is knocked-out, viral infectivity present in supernatants of infected fibroblast cultures is strongly reduced, thus driving virus dissemination in the cell monolayer towards a cell-associated pattern of focal spread. More recently, we found that mCMV forms an alternative gH/gL complex with MCK-2, the gene product of the mCMV m131–129 open reading frame (ORF). This trimeric gH/gL/MCK-2 complex facilitates infection of macrophages (MΦ) [18–19], a property also attributed to the pentameric gH/gL/pUL(128,130,131A) of HCMV *in vitro* [10,20]. The residual infectivity of gO-ko virus released into the supernatant of infected cells is MCK-2 dependent [18]. Besides associating with gH/gL complexes, both MCK-2 of mCMV and the UL128 protein of HCMV are also able to act as C-C chemokines attracting cells and modifying their functions [21–22]. In immunocompetent mice infected with MCK-2-ko mutants, reduced virus titers in salivary glands (SG), and reduced numbers of infected peripheral blood monocytes and tissue MΦ are observed [18,23–25]. Additionally, absence of MCK-2 is associated with a reduced recruitment of immunosuppressive inflammatory monocytes [26] and an enhanced anti-viral CD8 T-cell response [25,27]. It is currently not clear which of the observed phenotypes are due to MCK-2 functioning as a chemokine and which are due to, or modulated by, MCK-2 functioning as an entry mediator as part of the gH/gL/MCK-2 complex, nor whether these functions can be separated.

Here, we studied the *in vivo* host cell infection and subsequent intra-tissue spread of mCMV mutants selectively expressing either the gH/gL/gO or the gH/gL/MCK-2 complex, or lacking both of these alternative gH/gL complexes. We show that an efficient initial establishment of organ infection, with the notable

exception of SG infection, is crucially dependent on the gH/gL/gO complex. gO-transcomplementation of a genetic gO-ko mutant in virus ΔgO-gO^trans^ reversed the cell entry deficiency phenotype and, most notably, its gO-deficient progeny was then able to spread within different tissues with viral doubling times comparable to those of wild-type (WT) virus. This spread, however, required the alternative complex gH/gL/MCK-2, as revealed by absence of spread of viral progeny of the gO-transcomplemented double-ko mutant ΔgOΔMCK-2-gO^trans^.

In essence, these results revealed an example for a herpesvirus for which neither the gB and gH/gL core complexes alone, nor potentially unidentified other virion envelope glycoprotein complexes, can engender efficient infection *in vivo*. The alternative gH/gL/MCK-2 complex can substitute for the gH/gL/gO complex for intra-tissue spread and SG infection but not for entry into most first-hit target cell types in organs implicated in CMV disease.

## Results

### *In vivo* attenuation of mCMV mutants lacking gO is reversed by gO-transcomplementation

The gene product gO of mCMV ORF m74 forms a complex with the glycoproteins gH and gL. Deletion of m74, and thus of gO, in a recombinant virus is associated with a reduced infectivity of supernatant virus and a focal spread pattern in cell culture [17]. To investigate the role of gH/gL/gO, we used a recently described gO-ko mutant ΔgO (Δm74), which lacks 532 bp at the 5’ end of ORF m74 [18], and constructed an alternative ΔgO mutant (m74stop) containing a stop cassette that interrupts ORF m74 after 120bp. Both gO mutations were introduced into the genome of the mCMV Smith strain, cloned as a bacterial artificial chromosome (BAC), in which a preexisting m129/MCK-2 frameshift mutation was repaired [28]. Production of infectious virus in fibroblast cell cultures was reduced by a factor of ~100 when compared to WT virus (S1 Fig.). This reduction corresponded to a switch from wide-spread infection of the cell monolayer to focal spread (S2A Fig.). When spread via supernatant virus was experimentally inhibited by methylcellulose overlay, spread of WT virus was reduced to a focal spread pattern exhibiting foci comparable in size to foci formed by ΔgO mutants (S2B Fig.), whereas foci formed by ΔgO mutants were not altered. This indicated that short-distance spread between neighboring cells was not affected by the lack of gO. Neutralizing, but not non-neutralizing anti-gB antibodies, also rendered spread of WT virus focal and, furthermore, reduced the size of foci formed by WT virus as well as ΔgO mutants. (S2C–S2D Fig.). This finding suggests that focal spread involves transfer of released virions from the surface of infected cells to the surface of neighboring cells, a process accessible to antibodies. This conclusion is supported by *in vivo* inhibition of cell-to-cell spread of WT mCMV in liver tissue upon intravenous (i.v.) infusion of virus-neutralizing serum antibodies [29]. The residual spread in presence of neutralizing anti-gB antibodies likely reflects a component of direct cell-to-cell transmission in cell culture.

**Figure 1:**
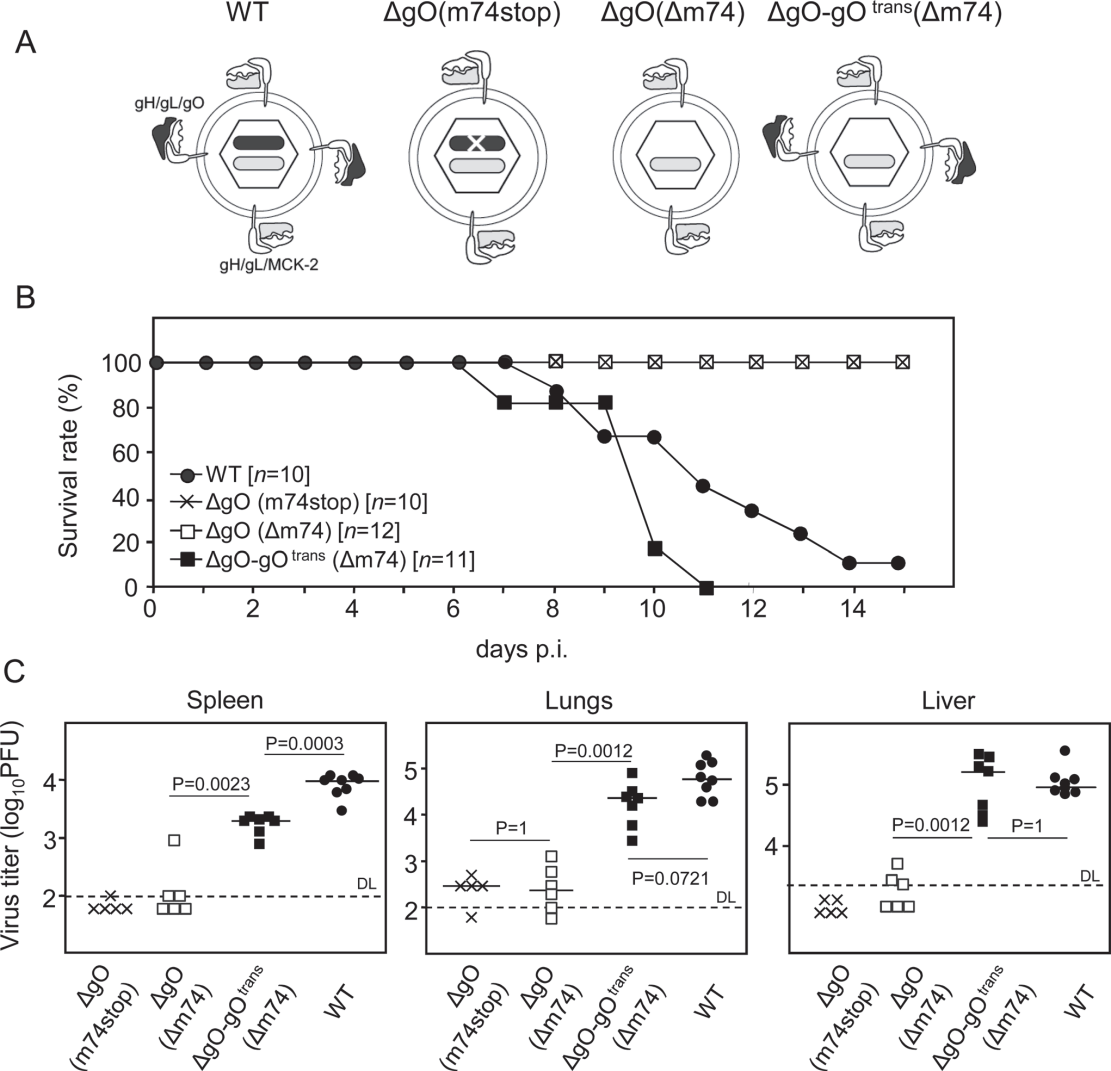
Knock-out of gO strongly impairs infection of otherwise highly-susceptible neonatal mice. (A) Virion pictograms illustrating alternative gH/gL complex envelope equipment of viruses used. Black bar within capsid symbol: ORF m74 encoding gO. Grey bar: ORF m131–129 encoding MCK-2. (B) Newborn BALB/c mice were infected i.p. with 2000 PFU of the indicated viruses and survival rates were monitored daily until day 15. (C) Newborn BALB/c mice were infected i.p. with 1000 PFU of the indicated viruses, and virus titers in organ homogenates (PFU/organ for spleen and lungs; PFU/g for the liver) were determined 9.5 days later. Symbols represent titers in organs of individual mice with median values marked. DL, detection limit. P values (distribution-free Wilcoxon-Mann-Whitney rank sum test, two-sided) for evaluating the significance of differences are indicated for group comparisons of most interest.

The thus characterized ΔgO mutants, in comparison to WT virus and a gO-transcomplemented virus ΔgO-gO^trans^ (virion pictograms in Fig. 1A), were then used to investigate the importance of the gH/gL/gO complex for virulence *in vivo*. gO-transcomplementation by propagation of ΔgO virus in gO-expressing cells (NIH-gO) generates phenotypically WT-like virions carrying gH/gL/gO complexes in the virion envelope available upon first entry into target cells [17–18]. In further rounds of infection, however, progeny of virus ΔgO-gO^trans^ are again ΔgO. This makes gO-transcomplementation an elegant approach to distinguish between gO requirement for first target cell entry and subsequent intra-tissue spread.

In a first set of experiments and first model situation (Fig. 1), we intraperitoneally (i.p.) infected newborn BALB/c mice known to be particularly susceptible to mCMV infection [30]. While mice infected with WT virus succumbed to CMV disease from day 7 onward, all those infected with either of the two ΔgO mutants survived (Fig. 1B). This indicated strong virulence attenuation of the mutants in clinical terms. Notably, virulence of ΔgO virus was restored and newborn mice died of CMV disease when gO was transcomplemented in virus ΔgO-gO^trans^, although gO was available only upon first cell entry. The survival/mortality rates corresponded to titers of infectious virus in diverse organs differing in cell type composition and tissue architecture, including spleen, lungs, and liver (Fig. 1C). Specifically, and consistently in all organs tested, virus titers with either of the ΔgO mutants were significantly lower than with WT virus or virus ΔgO-gO^trans^. These principles were essentially reproduced in a second model situation (S3 Fig.), the ‘immunocompromised host’ model involving i.v. infection of adult BALB/c mice after hematoablative total-body γ-irradiation (reviewed in [31]).

### Reversal of the growth deficiency phenotype of the gO-knockout results from gO-transcomplementation rather than from recombination

Since transcomplementation of genetic ΔgO virions with gO can only restore virus entry into the first-hit target cells, but not into neighboring cells in subsequent rounds of infection, reversion of the growth deficiency phenotype by gO-transcomplementation was an unexpected yet highly important result, as it revealed for the first time different molecular requirements for infection of first-hit target cells and subsequent intra-tissue spread. To exclude genetic recombination within the NIH-gO cells during propagation of virus ΔgO-gO^trans^, we tested the virion preparation for the absence of the deleted m74 sequence by qPCR. In addition, for excluding the possibility of an *in vivo* selection and expansion of trace amounts of recombined virus after infection with virus ΔgO-gO^trans^, we chose a two-color *in situ* hybridization (2C-ISH) strategy (S4A Fig.). Genomes from WT and mutant viruses are both stained red with hybridization probe m74.1 directed against the shared, undeleted region of m74, whereas absence of black stain after hybridization with probe m74.2 − specific for the 532-bp deletion in virus ΔgO − verified the genetic deletion status of the transcomplemented mutant. S4B Fig. shows 2C-ISH images for consecutive sections of liver tissue from mice infected with either WT virus or virus ΔgO-gO^trans^, or co-infected with both viruses. In none of the liver sections from mice infected only with the mutant virus ΔgO-gO^trans^ could the m74.2 sequence (black stain) be detected. This finding refutes the objection that recombination between the Δm74 mCMV genome and the gO-expressing vector used for transcomplementation might possibly have genetically restored ORF m74 in virus ΔgO-gO^trans^.

### Studies with cell lines predicted cell-type specific differences in the requirement of the gH/gL/gO complex

Since host tissues are composed of diverse cell types, we wondered if the observed *in vivo* attenuation phenotype of ΔgO mutants (Figs. 1 and S3) reflects a general cell entry deficit or a deficit in infecting particular cell types that account for most of the virus productivity, such as fibroblasts and epithelial cells. For a prediction from cell culture experiments, we infected cell lines representing fibroblasts (NIH3T3), epithelial cells (TCMK-1), EC (MHEC-5T), and MΦ (ANA-1) with WT mCMV, the two independent ΔgO mutants, and the gO-transcomplemented virus ΔgO-gO^trans^ (S5 Fig.). Compared to WT virus and normalized to infection of MEF (in which the viruses were grown and quantitated), both ΔgO mutants showed a reduced capacity to infect NIH3T3 fibroblasts and a loss of the capacity to infect TCMK-1 epithelial cells, phenotypes that were reverted by gO-transcomplementation. In sharp contrast, infection of ANA-1 MΦ was not affected and infection of MHEC-5T EC was even enhanced. Notably, enhanced infection of EC by ΔgO mutants was not reversed by gO-transcomplementation, a phenomenon that might be explained by non-physiological ratios of the alternative gH/gL complexes affecting the infection efficiency for EC [32–33]. In conclusion, the cell culture data predicted an entry deficit of ΔgO mutants for fibroblasts and epithelial cells but not for EC and MΦ.

### gO is required for an efficient initial virus entry into main cell types of the liver

**Figure 2:**
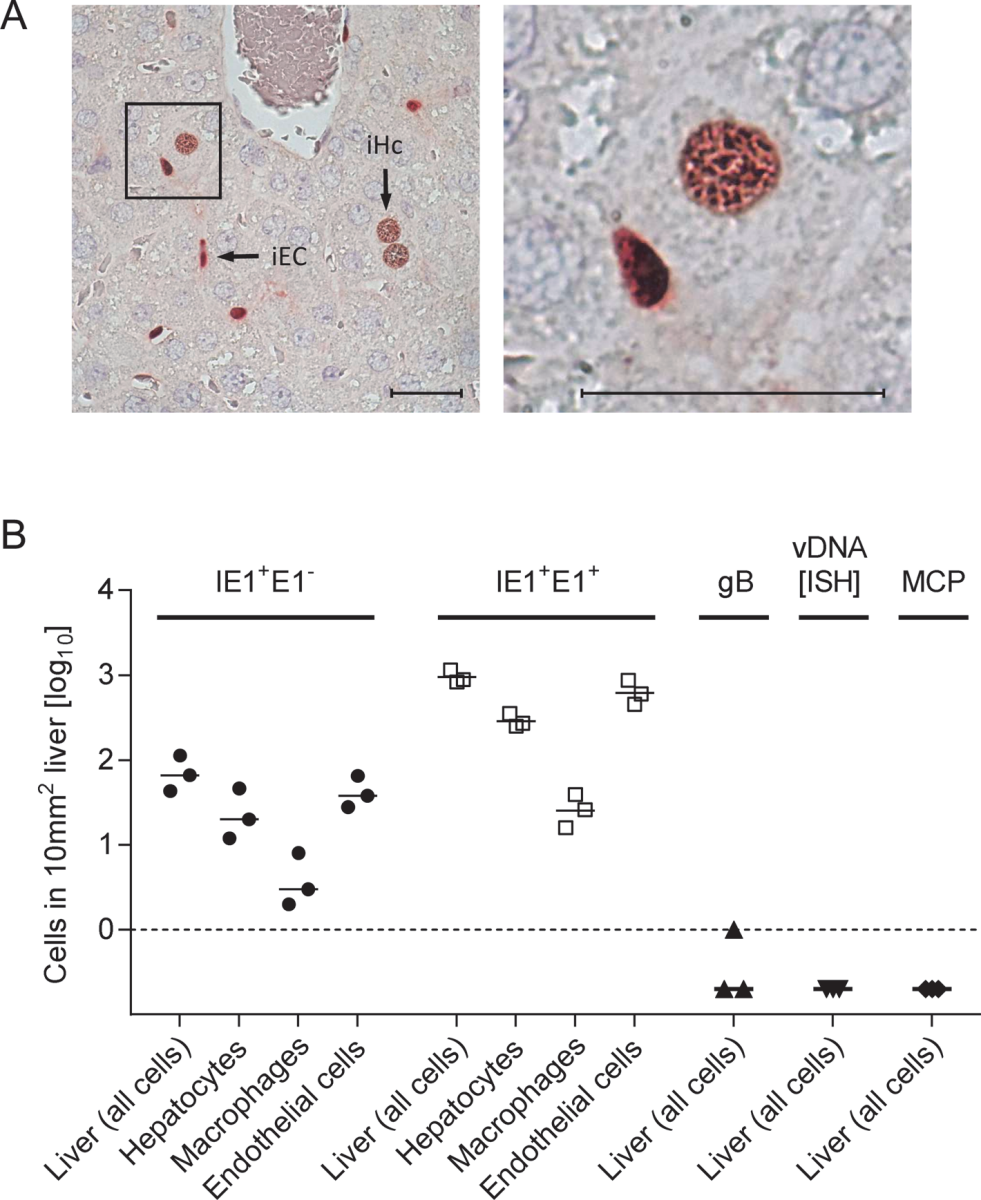
Synchronicity of infection initiation in main cell types of the liver. (A) 2C-IHC of liver tissue sections taken at 24h after i.v. infection of immunocompromised BALB/c mice (6.5 Gy of γ-irradiation) with 1 x 10^6^ PFU of WT mCMV, simultaneously detecting viral proteins IE1 (black staining) and E1 (red staining) in nuclei of infected cells. Left image: overview. iEC, infected endothelial cell; iHc, infected hepatocyte. The framed area is shown enlarged in the right image. Bar markers represent 25 μm. (B) Cell counts in representative 10-mm^2^ areas of liver tissue sections quantitating IE1^+^E1^-^ and IE1^+^E1^+^ cells differentiated by cell type as indicated. Infection had not proceeded in any cell type to expression of gB, viral DNA synthesis (vDNA detected by ISH), and the late (L) phase protein MCP. Symbols represent linked data from livers of 3 mice analyzed individually. The median values are marked.

Reduced virus titers measured several days after host infection can result from inefficient infection of first-hit target cells as the starting point, or from inefficient subsequent spread within tissue from initially infected cells to neighboring cells, or from a combination of both. For identifying and quantitating first-hit target cells *in vivo*, we used an approach allowing time too short for completion of the productive viral replication cycle, thus revealing the rate of cell entry uninfluenced by spread. For quantitating entry events without hindrance by immune defense on the route to target organs, we infected γ-irradiated mice i.v. (via the *vena cava inferior*) so that virus reaches its target tissues with the circulation within seconds, initiating an almost synchronized infection. Such a scenario has a clinical correlate in the early infection of patients conditioned by hematoablative treatment for a subsequent hematopoietic cell transplantation (HCT) (for a clinical review, see [34]). Under such defined conditions and at 24h after infection with WT mCMV, ~90% of the infected liver cells had proceeded to the second kinetic phase of viral gene expression, the early (E) phase, as indicated in 2-color immunohistochemical (2C-IHC) staining of intranuclear viral proteins immediate-early (IE)1 [35] and E1 [36–37] (IE1^+^E1^+^cells), whereas only ~10% of the cells expressed IE1 but not yet detectable amounts of E1 (IE1^+^E1^-^cells), indicating they were still in the IE phase (Fig. 2). Importantly, infected liver cells had not proceeded to expression of the essential glycoprotein gB (M55), replication of viral DNA, and expression of the essential late (L) phase major capsid protein MCP (M86), which proves that the first cycle was not completed and thus spread excluded (Fig. 2B).

**Figure 3:**
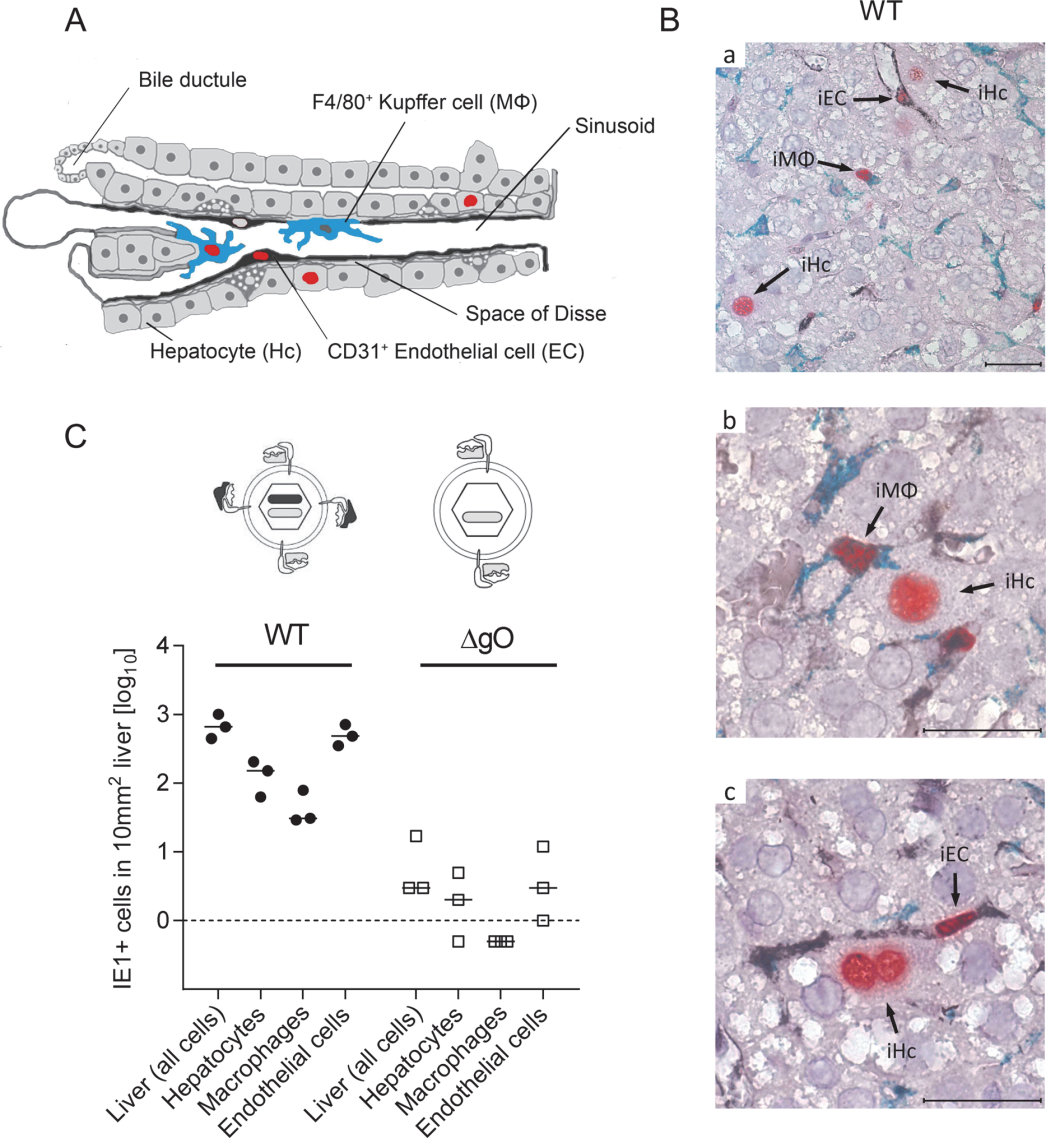
Requirement of gO for efficient initiation of infection in diverse liver cell types. (A) Sketch of liver tissue microanatomy with the localization of Hc, EC (black stain), and MΦ (turquoise-green stain). Infection of cells is symbolized by viral IE1 protein-containing cell nuclei (red stain). (B) 3C-IHC images of liver tissue sections taken at 24h after i.v. infection of immunocompromised BALB/c mice (6.5 Gy of γ-irradiation) with 1 x 10^6^ PFU of WT mCMV. (a) Overview showing infected Hc (iHc, red stained nucleus, IE1 protein), infected IE1^+^ (red) F4/80^+^ (turquoise green) MΦ (iMΦ), and infected IE1^+^ (red) CD31^+^ (black) EC (iEC). (b) Higher magnification image showing iMΦ and iHc in greater detail. (c) Higher magnification image showing iEC and a binucleated iHc in greater detail. Bar markers: 25 μm. (C) Counts of infected IE1^+^ cells (sum of IE1^+^E1^-^ and IE1^+^E1^+^ cells) of the indicated liver cell types in representative 10-mm^2^ areas of liver tissue sections after infection with viruses WT or ΔgO (Δm74) under the conditions specified above. Symbols represent data (linked data within each infection group) from individual mice with the median values marked.

With a focus on the liver, for quantitating successful entry events differentiated by liver cell type, we combined IHC detection of the IE1 protein with cell type-specific markers (Fig. 3). In the liver sinusoids (for a sketch, see Fig. 3A; modified from [38]), virions directly meet CD31^+^ liver sinusoidal endothelial cells (LSEC), which form the lining of the sinusoids and are noted targets of acute [39] and latent [40] mCMV infection. Virions also directly meet liver-resident F4/80 (Ly71)^+^ MΦ, known as Kupffer cells, which localize to the sinusoidal lumen attached to the sinusoidal lining and are also recognized targets of mCMV infection [18]. Although hepatocytes (Hc) are separated from the sinusoidal lumen by the fenestrated endothelium and the space of Disse, they are also first-hit target cells of mCMV because virions can pass through the fenestrae. This has originally been indicated by detection of recombined rec-egfp virus in Hc within 24h after infection of Alb-cre mice with floxed reporter virus. Reciprocally, Hc were infected with unrecombined reporter virus 24h after infection of Tie2-cre mice, in which rec-egfp virus was still confined to EC [39].

A three-color IHC (3C-IHC) approach distinguished between infected Hc (IE1^+^ iHc, red nuclear staining), which are distinctive by cytomorphology, infected MΦ (IE1^+^F4/80^+^ iMΦ, red and turquoise-green), and infected EC (IE1^+^CD31^+^ iEC, red and black) (Fig. 3B). Most cells expressing IE1 at 24h after infection with WT virus were EC, followed by Hc and MΦ (Figs. 2B and 3C). As shown in Fig. 2B, most of the IE1^+^ cells co-expressed protein E1, indicating they were in the E phase. Remarkably, this applied to all three cell types, revealing for the first time synchronicity of *in vivo* viral gene expression in EC, MΦ, and Hc despite their different localization in the tissue. The ranking of the cell types in the absolute numbers of infected cells might reflect their quantitative representation in the liver; alternatively, it might also reflect cell-type specific differences in the cells’ propensity to become infected. To answer this question, we related the numbers of infected cells of each cell type to the corresponding numbers of all cells (S6 Fig.). Whereas the percentages of iHc and iMΦ were ~1%, the percentage of iEC was significantly higher, namely ~5%. This does not necessarily indicate a higher susceptibility of EC to infection in molecular terms; rather, this might reflect a better accessibility of EC that—by lining the sinusoids—provide a huge surface ideal for virion entry.

Importantly, absence of gO in virus ΔgO substantially reduced the number of infected cells, and this consistently applied to all three cell types (Fig. 3C), demonstrating that gH/gL/gO is critical for efficient virus entry into quite diverse cells. It may be of interest to note that co-infection with WT and ΔgO viruses did not inhibit WT virus infection, a finding that excludes the possibility of ΔgO defective particle interference in the entry process or enhanced innate/intrinsic defenses elicited by high numbers of ΔgO particles as alternative explanations for poor infectivity of ΔgO viruses.

Since ΔgO viruses still carry the alternative complex gH/gL/MCK-2, the data imply that gH/gL/MCK-2 is not an efficient entry mediator on its own, although our previous work has shown that gH/gL/MCK-2 improves the efficacy of entry, specifically into F4/80^+^ liver MΦ, in viruses co-expressing gH/gL/gO [18]. Altogether, with respect to cell entry of virus arriving from the circulation, gH/gL/MCK-2 cannot substitute for gH/gL/gO. Strikingly, the requirement of gH/gL/gO for cell entry *in vivo* applied to all main cell types of the liver. This finding was not predicted by the specific cell lines used for the *in vitro* studies (recall S5 Fig.).

### Intra-tissue virus spread proceeds virtually unabated also in the absence of gO

A reduced increase in virus titers in organs over time may be due to an impaired virus spread within the respective organ or to reduced initial numbers of infected cells. To understand the problem, one must consider that virus multiplication follows an exponential function, so that lower initial numbers of infected cells develop into increasing differences in absolute titers over time, even when the viral capacity for spread within tissue, which is characterized by the virus doubling time (vDT), is actually unaffected by the mutation under study. Exponential functions are linearized by log-transformation of the measured values of the dependent variable, the Y-axis values, to make the data accessible to linear regression analysis. This allows calculation of vDT from the slope of the regression line (reviewed in [41]). In essence, in a comparison of two viruses, parallel regression lines indicate identical spread capacities, whereas diverging regression lines indicate different spread capacities.

**Figure 4:**
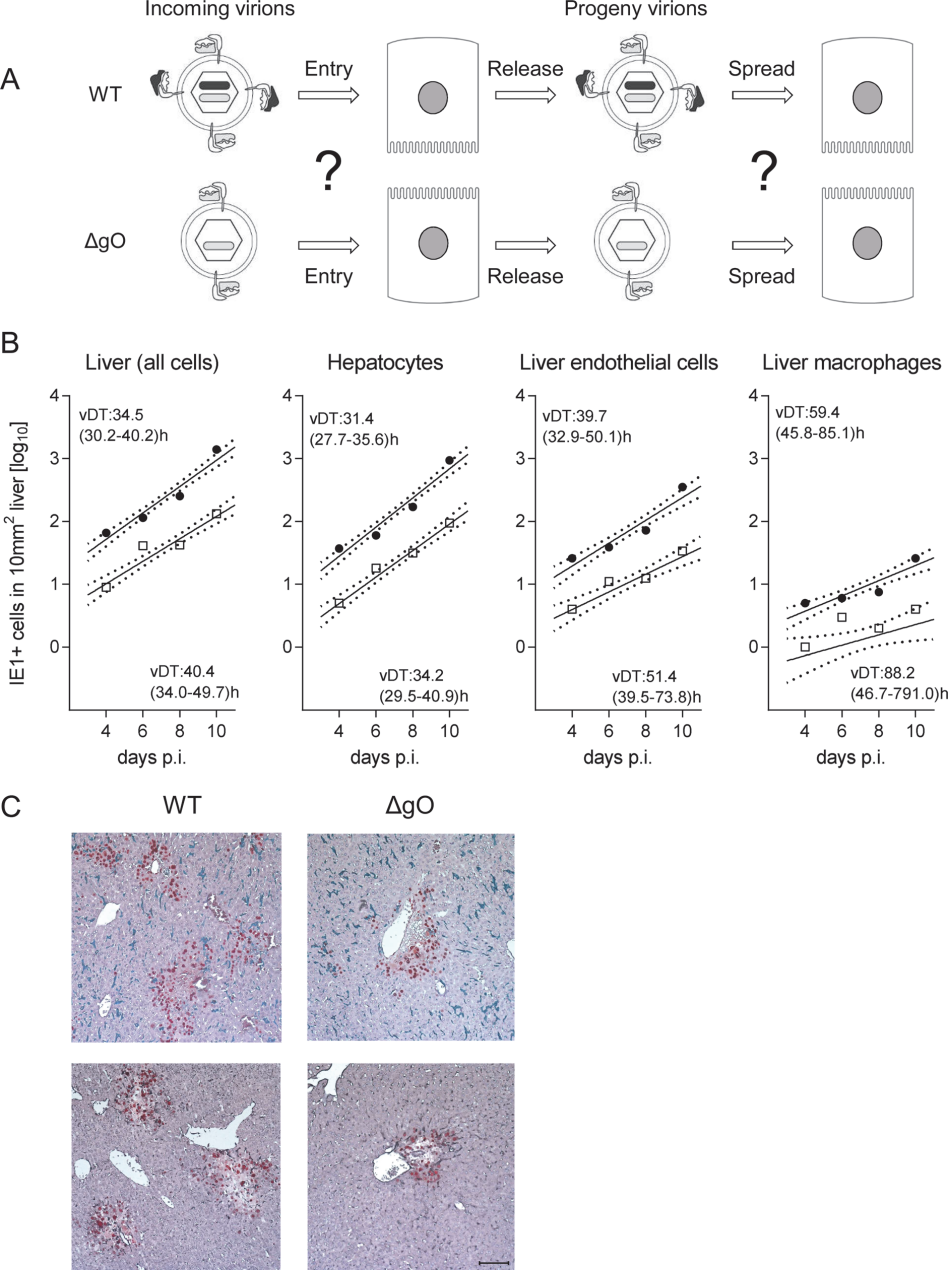
gO-independence of virus spread in liver tissue. (A) Sketch of the concept with WT and ΔgO (Δm74) virion pictograms explaining the gH/gL complex envelope equipment of viruses upon first cell entry (incoming virions) and of their progeny participating in subsequent intra-tissue spread. (B) Time course of counts of infected IE1^+^ liver cells, all cells or differentiated by cell type, after i.v. infection of immunocompromised BALB/c mice (6.5 Gy of γ-irradiation) with 10^3^ PFU of WT virus (filled circles) or ΔgO virus (open squares). Symbols represent the median values of cell counts per representative 10-mm^2^ areas of liver tissue sections from at least 3 mice per group and time of assay. Log-linear regression lines (based on all data) and their corresponding 95% confidence areas (bordered by dotted lines) are indicated. Viral doubling times (vDT) were calculated based on the slopes *a* of the regression lines according to the formula vDT = log2/*a*. The 95% confidence intervals of vDT are given in parentheses. (C) 2C-IHC images taken on day 10 after infection with WT virus (left panels) or ΔgO virus (right panels). Upper two images show representative tissue section areas stained for IE1 (red) and the macrophage marker F4/80 (turquoise green). Lower two images show representative tissue section areas stained for IE1 (red) and the EC marker CD31 (black). The bar marker represents 100 μm and applies to all 4 images.

After infection with WT virus, gH/gL/gO is available for first entry and for spread, whereas after infection with ΔgO it is unavailable throughout (Fig. 4A). Comparing these two viruses for growth in the liver resulted in fairly parallel log-linear regression lines, though with 1-log distance from each other in numbers of infected cells at any time, which reflects the known difference in initial infection (recall Fig. 3) followed by an almost equally efficient intra-tissue spread (Fig. 4B, outer left panel). Analysis of specific liver cell types (Fig. 4B, remaining panels) revealed almost identical vDT for WT and ΔgO virus in Hc, whereas there is a trend to somewhat slower spread of the mutant among EC and MΦ. The generally poor spread in MΦ likely relates to the fact that MΦ are solitary cells not establishing firm contacts with each other or with other cell types, which hampers virus transfer from cell to cell, whereas Hc form a three-dimensional parenchyma with intimate cell contacts. It has to be taken into account that virus spread occurs not only between cells of the same type but also between different cell types, as was documented previously by bidirectional spread between Hc and EC [38]. The conclusion that spread is unaffected or only minimally affected by deletion of gO is visually confirmed by IHC images directly showing a lower number but comparable size of ΔgO foci (Fig. 4C).

### gO-transcomplementation reverts the entry deficit of ΔgO virus and restores virus growth and histopathology

**Figure 5:**
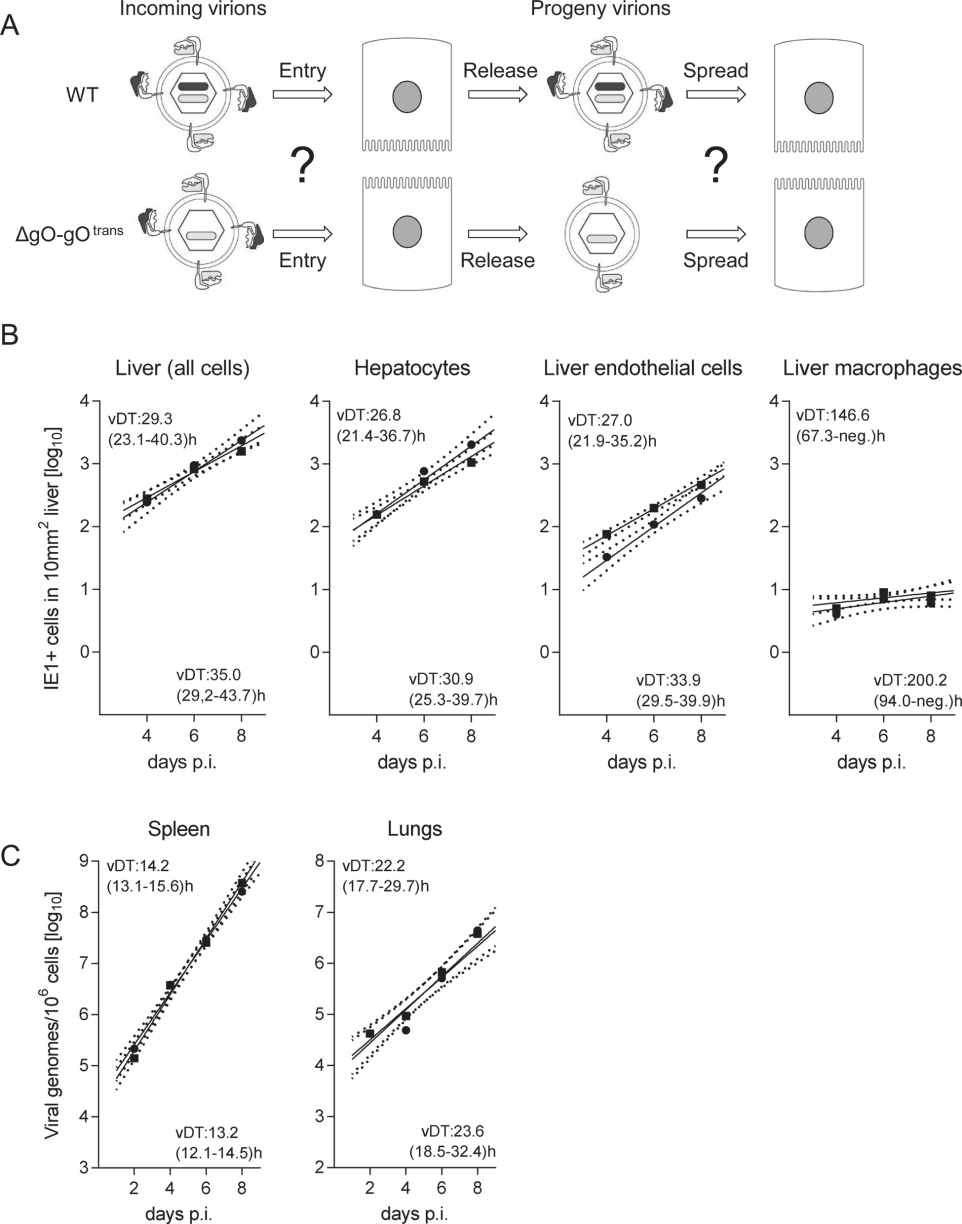
Reversal of the ΔgO growth deficiency phenotype by gO-transcomplementation. (A) Sketch with WT and ΔgO-gO^trans^ virion pictograms explaining the gH/gL complex envelope equipment of viruses upon first cell entry (incoming virions) and of their progeny participating in subsequent intra-tissue spread. (B) Time course of counts of infected IE1^+^ liver cells, all cells or differentiated by cell type, after i.v. infection of immunocompromised BALB/c mice (6.5 Gy of γ-irradiation) with 10^3^ PFU of WT virus (filled circles) or virus ΔgO-gO^trans^ (filled squares). Symbols represent the median values of cell counts per representative 10-mm^2^ areas of liver tissue sections from at least 3 mice per group and time of assay. (C) Corresponding analysis of viral DNA load in spleen and lungs (mean of triplicate tissue samples per mouse) by qPCR specific for gene M55 (encoding gB), with qPCR specific for cellular gene *pthrp* performed for normalization to host cell numbers. Symbols represent median values from at least 3 individually tested mice per group and time of assay. For the explanation of log-linear regression analysis (calculating vDT), see the legend of Fig. 4.

**Figure 6:**
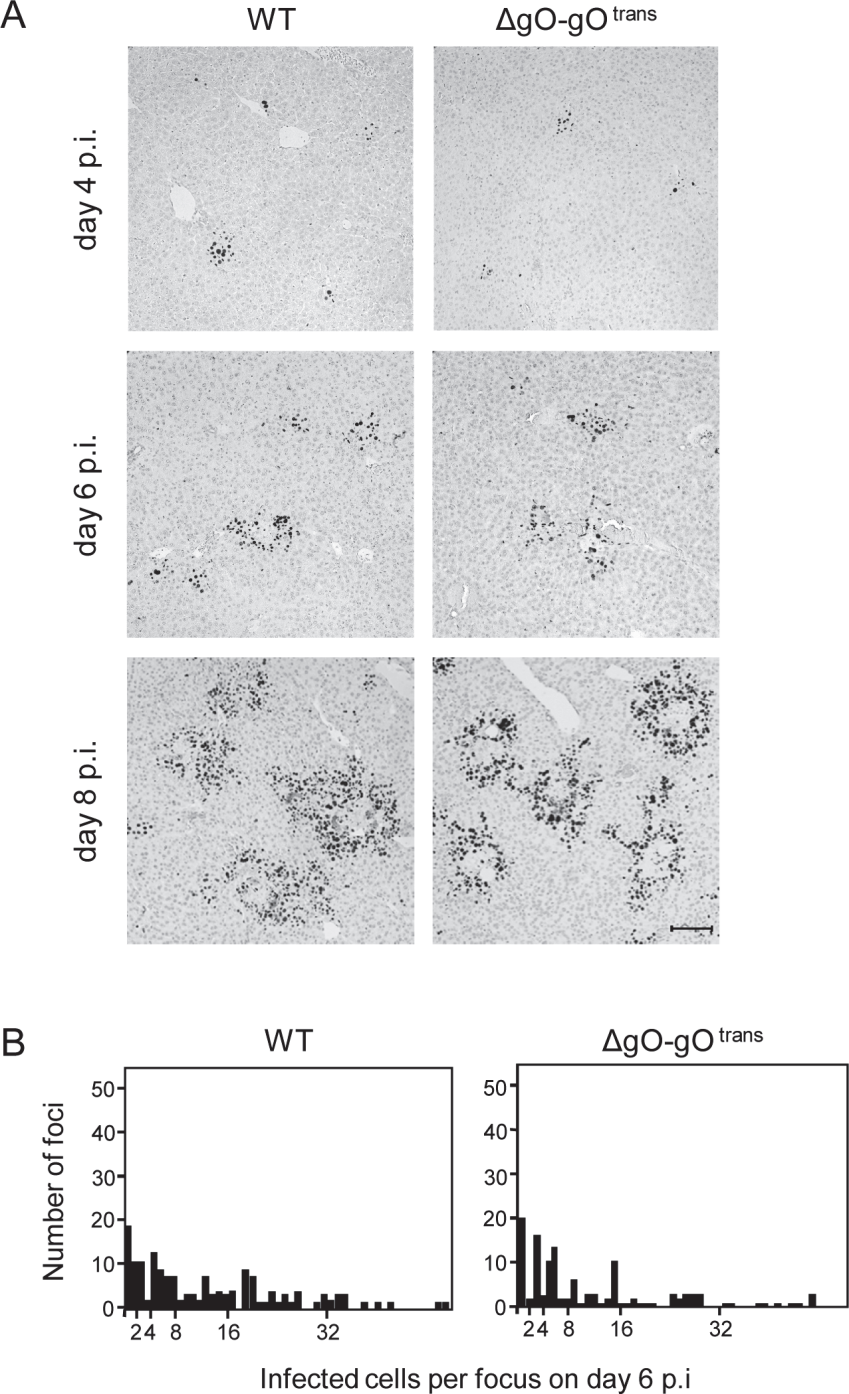
Comparable development of infection foci of viruses WT and ΔgO-gO^trans^. Immunocompromised BALB/c mice (6.5 Gy of γ-irradiation) were infected i.v. with 10^3^ PFU of WT virus or of virus ΔgO-gO^trans^. (A) IHC images of liver tissue sections were taken on days 4, 6, and 8 p.i. to show the growth development of viral foci over time. Infected cells are visualized by black staining of intranuclear IE1 protein. Bar marker: 100 μm. (B) Bar diagrams of the focus size distributions on day 6 for representative 10-mm^2^ areas of liver tissue sections, revealing similar numbers and comparable sizes of foci of infection for the two viruses under study.

Virus ΔgO-gO^trans^ carries the gH/gL/gO complex upon first cell entry, but its progeny are ΔgO again (Fig. 5A). Notably, unlike the situation seen above for ΔgO, comparing viruses WT and ΔgO-gO^trans^ for growth in the liver now revealed superposable regression lines with regard to all liver cells as well as to the individual liver cell types (Fig. 5B), suggesting equivalent growth properties in terms of both initial entry and subsequent spread. Alternatively, identical numbers of infected liver cells could have resulted from many small foci of infection with one of the viruses and fewer but larger foci for the other. This alternative explanation is refuted, however, by IHC images of liver tissue sections demonstrating comparable numbers and size distributions of infectious foci for the two viruses in the time course (Fig. 6).

To test if the same rules apply also to other organs involved in CMV disease, we determined log-linear growth regression lines in spleen and lungs by quantitating the increase in viral genome load over time, and found identical growth of WT and ΔgO-gO^trans^ (Fig. 5C). In conclusion, despite marked differences to the liver in terms of cell type composition and overall tissue architecture, virus spread can proceed in the absence of gH/gL/gO also in organs other than the liver.

### The alternative complex gH/gL/MCK-2 is required for intra-tissue virus spread only in the absence of gH/gL/gO

gO-independence of intra-tissue virus spread suggested involvement of an alternative viral envelope glycoprotein complex. Although infection of most organs has been shown not to depend on MCK-2 when gH/gL/gO is present ([23,24] and S7 Fig.), it remained possible that spread can be promoted in a redundant fashion by either gH/gL/gO or gH/gL/MCK-2. If that was true, co-deletion of both complexes should strongly diminish virus spread within organs. A first hint for such a function of MCK-2 was given by data on viral spread in fibroblast cell cultures (S8 Fig.). As double-ko virus ΔgOΔMCK-2 does not produce infectious progeny in cell culture [18], it was necessary to transcomplement gO. When compared to virus ΔgO-gO^trans^ still carrying the alternative gH/gL/MCK-2 complex (S8 Fig., left images), foci from ΔgOΔMCK-2 progeny of ΔgOΔMCK-2-gO^trans^ virus barely expanded (S8 Fig., center images), indicating an involvement of gH/gL/MCK-2 in viral spread *in vitro*. This conclusion was further corroborated by inhibition of viral spread in the presence of antibodies directed against MCK-2 (S8 Fig., right images).

**Figure 7:**
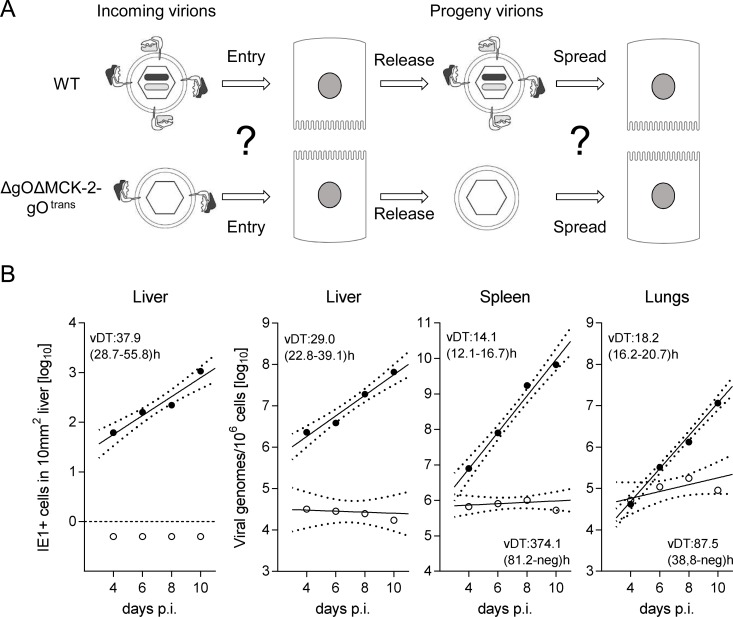
Double-ko of gO and MCK-2 ablates *in vivo* virus growth. (A) Sketch with WT and ΔgOΔMCK-2-gO^trans^ virion pictograms explaining the gH/gL complex envelope equipment of viruses upon first cell entry (incoming virions) and of their progeny participating in subsequent intra-tissue spread. (B) Time course of counts of infected IE1^+^ liver cells (outer left panel) or of qPCR-determined viral genome loads in liver, spleen, and lungs (remaining panels) after i.v. infection of immunocompromised BALB/c mice (6.5 Gy of γ-irradiation) with 10^3^ PFU of WT virus (filled circles) or ΔgOΔMCK-2-gO^trans^ virus (open circles). For the explanation of log-linear regression analysis (calculating vDT), see the legend of Fig. 4.

Since *in vitro* foci from virus ΔgOΔMCK-2-gO^trans^ still existed, though substantially condensed in size, we asked how simultaneous absence of both alternative gH/gL complexes would translate to virus growth *in vivo* in liver, spleen, and lungs (Fig. 7). In the liver, the number of cells infected by ΔgOΔMCK-2 progeny of ΔgOΔMCK-2-gO^trans^ virus remained below the detection limit and the number of viral genomes from the initial infection even slowly declined over time, thus indicating complete absence of intra-tissue spread. Spread was also undetectable in the spleen, whereas one might discuss some residual − yet very inefficient − spread in the lungs.

### Salivary gland infection does not follow the rule

**Figure 8:**
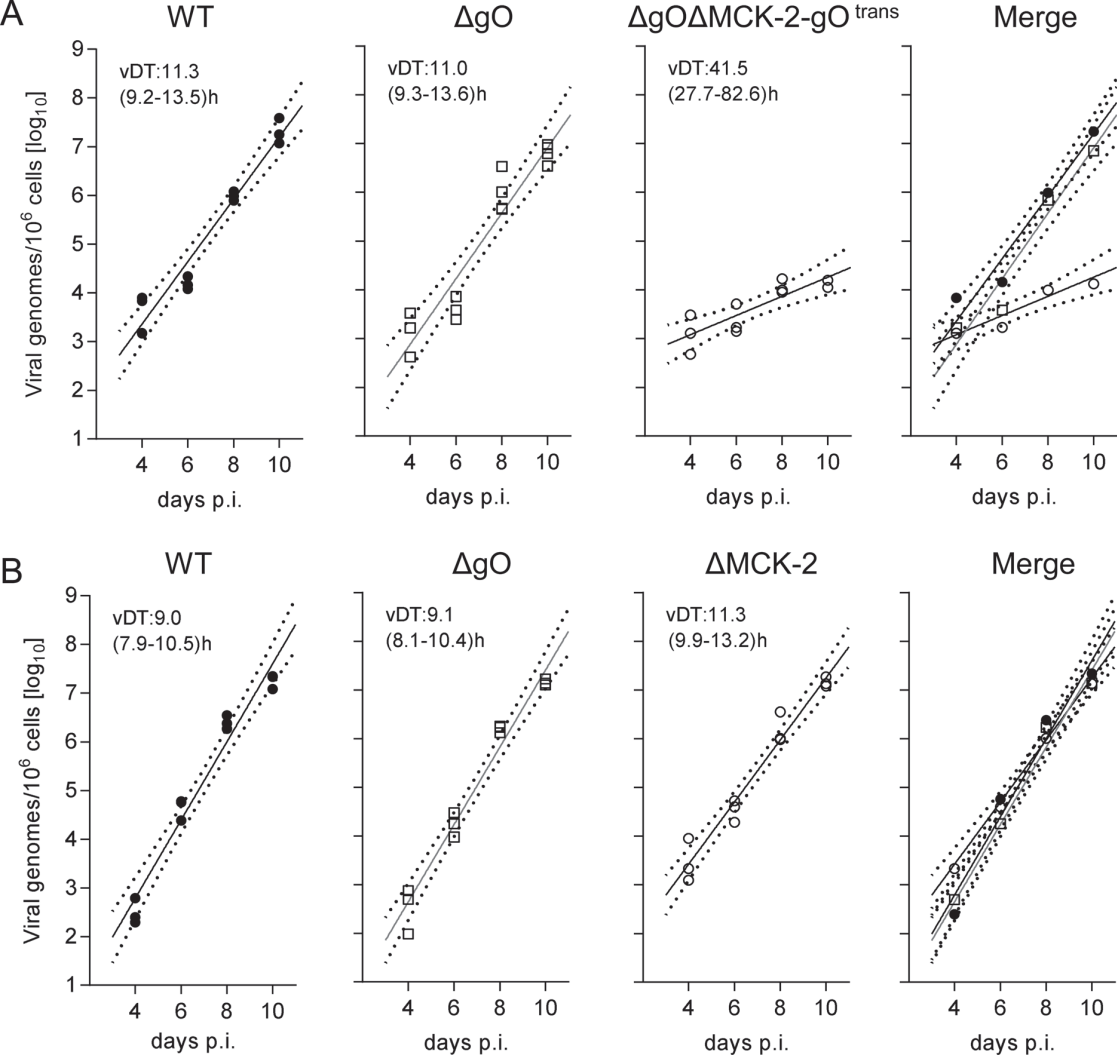
Redundance of alternative gH/gL complexes gH/gL/gO and gH/gL/MCK-2 in securing the infection of salivary glands. (A) Time course of SG infection (for conditions and qPCR assay see the legend of Fig. 5) by viruses WT (filled circles), ΔgO (open squares), and ΔgOΔMCK-2-gO^trans^ (open circles). (B) Independent second experiment reproducing the time course of SG infection by viruses WT (filled circles) and ΔgO (open squares), now compared to virus ΔMCK-2 (open circles) still expressing the gH/gL/gO complex. Symbols in the three single virus panels represent data from individual mice, symbols in the merge (outer right) panel represent the corresponding median values. For the explanation of log-linear regression analysis (calculating vDT), see the legend of Fig. 4.

An unexpected result was obtained by the analysis of gH/gL complex requirements for the infection of SG (Fig. 8). Unlike what was seen for the other organs, even uncomplemented ΔgO virus replicated like WT virus (Fig. 8A), indicating gO-independence of entry into glandular epithelial cells, the main virus-producing cell type in SG [42]. Notably, SG infection by ΔgO virus apparently depended on the expression of MCK-2, since double deletion in virus ΔgOΔMCK-2-gO^trans^ strongly reduced SG infection, resulting in a 3-log difference in the viral genome number on day 10 compared to WT and ΔgO virus (Fig. 8A). From these findings it was tempting to conclude that the gH/gL/gO complex is not involved in either step of SG infection and that instead the gH/gL/MCK-2 complex plays a non-redundant, essential role.

This interpretation, however, was corrected by an independent experiment comparing WT and ΔgO virus with a ΔMCK-2 virus lacking the gH/gL/MCK-2 complex but still expressing the gH/gL/gO complex (Fig. 8B). Surprisingly, ΔMCK-2 virus replicated in the SG like WT and ΔgO virus, indicating that MCK-2 is not essential but can be substituted by gO.

In conclusion, the alternative gH/gL complexes gH/gL/gO and gH/gL/MCK-2 mediate efficient viral growth and cannot be substituted in their roles by any other virion envelope glycoprotein complexes. Whereas gH/gL/gO and gH/gL/MCK-2 mediate intra-tissue spread as well as SG infection in a redundant fashion capable of replacing each other, gH/gL/gO is essential for entry into first-hit target cells in most organs, with the notable exception of the SG.

## Discussion

For HCMV, two gH/gL complexes, gH/gL/gO and gH/gL/pUL(128,130,131A), were characterized and corresponding target cells identified *in vitro* (see the Introduction). *In vivo* identification of infected cell types is usually based on autopsy or biopsy material derived from immunocompromised patients with overt disease [43], so that one cannot distinguish between first-hit target cells and secondarily infected cells, and virus intra-tissue spread in a time course is difficult, if not impossible, to assess in humans, as it would require repeated biopsies in patients. Due to the host restriction of CMVs, *in vivo* studies with viruses mutated in their outfit with envelope glycoprotein complexes are limited to natural host animal models, of which infection of mice with mCMV is the most intensively explored. For mCMV, also two gH/gL complexes, gH/gL/gO and gH/gL/MCK-2, are known [17–18]. Depending on route of infection and immune status, local fibrocytes, MΦ in lymphoid tissues and liver, EC, Hc as an epithelial cell type, and more recently also mast cells [44] and alveolar MΦ [19] are noted first-hit target cells of mCMV [39,45].

Cell culture analyses of HCMV and mCMV ΔgO mutants in fibroblasts agreed in demonstrating a markedly reduced infectivity of virus progeny and a focal spread pattern [13,14,17]. A critical requirement for the mCMV gH/gL/gO complex was here also documented for entry into cells of the epithelial cell line TCMK-1. We expanded on these insights with the primary objective to identify the *in vivo* role of gO with the aim to confirm the predictions. In accordance with the cell culture studies, we found that ΔgO mutants of mCMV are indeed strongly attenuated in virulence in terms of virus replication and pathogenesis in vital organs. In contrast, infection of the EC cell line MHEC-5T and of ANA-1 MΦ revealed gO-independence *in vitro*, a prediction from cell culture that did not hold true *in vivo* for liver EC and MΦ, the infection of which proved to be gO-dependent.

By following an experimental strategy to distinguish between first virus entry into liver cell types and subsequent intra-hepatic virus spread, we found that absence of gO strongly reduced the numbers of initially infected EC, MΦ, and Hc. In contrast, the capacity for subsequent intra-hepatic spread, as reflected by virus doubling times (vDT), was unaffected by the mutation. These rules applied also to spleen and lungs. For the lungs, this finding is remarkable in that − for entering lung parenchyma from the circulation − virus has to pass EC that form a barrier of continuous lung endothelium [39], a step during which trans-complemented gO is necessarily lost. This indicates gO-independent spread from pulmonary vascular EC to interstitial cells and alveolar epithelium [44]. Thus, like in the liver, the first cell entry after arrival via the circulation proved to be the gO-dependent critical step for organ infection.

In our model of i.v. liver infection after hematoablative treatment, not only cells directly accessible from the lumen of the sinusoids, such as EC and MΦ, but also Hc, which are separated from the circulation by the fenestrated sinusoidal endothelium, became infected synchronously without a preceding viral replication cycle in any other cell type. This argues against a general involvement of an interposed hematopoietic cell type on the route to target tissues. An exception appears to be the long-distance virus dissemination to the SG. Previous work has already indicated a special situation for the SG − distinguishing infection of SG from that of other organs − in that mCMV does not appear to reach this site as free virions but needs to hijack CX3CR1^hi^ patrolling monocytes (PM) to serve as vehicles transporting it to the SG, a mechanism that was concluded to depend on MCK-2 [21,25]. In this context, our data add the important new information that virus entry into glandular epithelial cells, the main virus-producing cell type in SG, can take place independently of gO, because MCK-2 can substitute for gO in its role. The result that, in our infection model, gO can in turn substitute for MCK-2 in SG infection, as seen with virus ΔMCK-2, was quite unexpected in view of previous work in immunocompetent mice having documented an SG growth deficiency of virus mutants not expressing MCK-2 [18,46–47]. A first hint for a model-dependent difference was given by the previous finding that the prototype of BAC-cloned mCMV [48]), in which an m129/MCK-2 frameshift mutation prevents the expression of full-length MCK-2 [49], replicated like WT virus in the SG of γ-irradiated mice even after local, intraplantar infection [30]. This argues against a critical role of the route of infection and leaves the hematoablative treatment as the differential parameter. Future studies will be aimed at identifying the hematopoietic cell that, in immunocompetent mice, restricts virus dissemination to the SG making it dependent on MCK-2. The PM discussed above [21,25] is an obvious candidate.

Interestingly, horizontal host-to-host transmission occurs through free monocapsid virions released with packed vacuoles from the glandular epithelial cells into the salivary duct [42] and thus the need for an efficient docking of free virions to first-hit target cells may have been the evolutionary driver for the acquisition of the gH/gL/gO complex.

The apparent question remains why intra-tissue spread does not require the gH/gL/gO complex. There exist examples for other viruses indicating that infection of cells by free virions arriving at first target cells can differ from the transfer of virus from an infected cell to closely neighboring cells. Mechanisms discussed for cell-to-cell spread include the formation of polarized contacts, so-called ‘virological synapses’ [49–50], transit through cell junctions [51–53], and transfer of extracellular virus involving the formation of membrane protrusions [54]. These modes of cell-to-cell transfer have in common a high local virus concentration associated with a high efficiency of infection and may differ from infection by free virions in the requirements for virion constituents [50]. Interpreting our findings, we propose that sufficient avidity for the binding of free virions to target cells depends on the gH/gL/gO complex, whereas for cell-to-cell spread either of the alternative gH/gL complexes is sufficient so that gH/gL/MCK-2 can substitute for gH/gL/gO in the spread of ΔgO viruses. If one of the alternative gH/gL complexes is preferentially used during spread of WT virus is open to question and might also depend on the relative amounts of these complexes incorporated into the virion envelope. These may vary with the cell type in which the virus has replicated [16] and may also differ between virus strains [32–33].

As alternative gH/gL complexes of HCMV strains cannot be studied *in vivo* with respective mutants, it must necessarily remain speculative whether our findings in the mCMV model exactly predict the roles the corresponding alternative gH/gL complexes play in human infection. In cell culture, the gH/gL/gO complexes of HCMV and mCMV were functionally comparable [13,14] and reduced capacities to infect MΦ apply to both, HCMV lacking gH/gL/pUL(128,130,131A) and mCMV lacking gH/gL/MCK-2 [10,18]. Reduced capacity of gH/gL/pUL(128,130,131A) deletion mutants of HCMV to infect EC and epithelial cells *in vitro* [6], was not seen with mCMV mutants lacking the gH/gL/MCK-2 complex [18]. Yet, data on cell tropism found in cell culture need not necessarily extrapolate to *in vivo* cell tropism. Cells in cell culture, and in particular immortalized and often clonal cell lines, likely differ in many respects from cells of the same cell types *in vivo*, and also cell contacts and cytokine milieu in cell monolayers do not always reflect those in the context of tissues. Our own data on EC and MΦ cell lines MHEC-5T and ANA-1, respectively, showed gO-independence of the infection, which did not apply to *in vivo* infection of these two cell types in the liver. Similarly, recent findings showed that mast cells *in vivo*, but not cultured mast cells, are susceptible to productive mCMV infection [44,55], and that PM, but not inflammatory monocytes, are infected by mCMV *in vivo*, although MΦ derived from both monocyte populations were found to be equally susceptible *in vitro* [25].

In summary, this first report on the roles alternative gH/gL complexes of a CMV play *in vivo* shows redundance in mediating intra-tissue virus spread as well as infection of SG, the site of virus host-to-host transmission, and revealed a critical role for gO in the initiation of infection by free virions. This makes the gH/gL/gO complex an interesting target for prevention of primary infection.

## Materials and Methods

### Mice and infection conditions

BALB/c mice were bred and maintained under SPF conditions at the Laboratory Mouse Breeding and Engineering Centre of the Faculty of Medicine, University of Rijeka, or in the Central Laboratory Animal Facility at the University Medical Center Mainz.

For immunosuppression, hematoablative conditioning of 8–9 week-old female BALB/c mice was achieved by total-body γ-irradiation with a single dose prior to infection. Adult mice were infected i.v. with tissue culture (NIH3T3)-derived mCMV WT or mutants in 500 μl of PBS. Neonatal mice were infected i.p. with the indicated viruses in 50 μl of PBS at 6 h post-partum. All mice were sacrificed by CO_2_ inhalation or cervical dislocation.

### Ethics statement

Animal research protocols of the University Medical Center Mainz were approved by the ethics committee of the Landesuntersuchungsamt Rheinland-Pfalz, permission no. 23 177–07/G09–1–004, according to German Federal Law §8 Abs. 1 TierSchG (animal protection law). All experiments done at the University of Rijeka, Croatia, were in accordance with the University of Rijeka animal use and care policies in accordance to the guidelines of the animal experimentation law (SR 455.163; TVV) of the Swiss Federal Government.

### Cells, viruses, and studies in cell culture

Primary mouse embryonic fibroblasts (MEF) from BALB/c mice, NIH3T3 cells (ATCC: CRL-1658), the endothelial cell line MHEC5-T [56] and the epithelial cell line TCMK-1 (ATCC: CCL-139) were maintained in Dulbecco’s modified Eagle’s medium (DMEM) supplemented with 10% fetal calf serum. The macrophage cell line ANA-1 [57] was maintained in RPMI medium supplemented with 10% fetal calf serum. NIH3T3 cell lines stably expressing m74/gO (NIH-gO) were used for gO-transcomplementation [17]. BAC (pSM3fr-MCK-2fl)-derived virus [28] was used as WT mCMV. The mCMV ORF m74 deletion mutant (ΔgO) and the m74/m131–129 double-knockout mutant (ΔgOΔMCK-2) have been described previously [18]. For analysis of virus spread and dissemination in cell culture, MEF were seeded in flat-bottomed 96-well plates and cell monolayers were infected with 50 PFU per well. One hour after infection, cell monolayers were washed and incubated for further 3 days with culture medium supplemented, depending on the question, with neutralizing anti-gB antibody (mAb, clone 97.3) [58], non-neutralizing anti-gB antibody (mAb, clone 5F12; kindly provided by Michael Mach, University Erlangen-Nürnberg, Germany), or rabbit anti-MCK-2 serum WU1073 [59]. Foci of infection or percentages of infected cells were visualized by indirect immunofluorescent staining of mCMV gB protein using anti-gB mAb (clone 97.3), or of mCMV intranuclear IE1 protein pp89/76 using mAb CROMA101. For counterstaining of cell nuclei, cells were incubated in PBS containing 5 μg/ml Hoechst 333258 (Invitrogen). To monitor virus infection of cells in suspension, intracellular cytofluorometric staining was performed. Briefly, cells were detached with 0.5 mM Na-EDTA, fixed with 1% paraformaldehyde for 10 min, and then stained in PBS containing 0.3% Saponin and 1% BSA using anti-IE1 antibody and Fluor488-coupled goat anti-mouse antibody (Invitrogen). Cells were washed with PBS containing 0.03% Saponin. After staining, cells were resuspended in 1% paraformaldehyde and analyzed on a FACSCalibur using CellQuest software (BD Biosciences).

### BAC mutagenesis and reconstitution of recombinant virus

Markerless BAC mutagenesis was performed to introduce a stop cassette in the m74 ORF in the pSM3fr-MCK-2fl BAC as described previously [60]. For constructing the pSM3fr-m74stop mutant (virus: ΔgO; m74stop), the primers m74stop-for (5’-GGA GGT TCG GTC GCA TCG ATT GTA TCA TAA CCT CCG TCT TCA TAA TCA TC*G GCT AGT TAA CTA GCC* AGG ATG ACG ACG ATA AGT AGG G-3’) and m74stop-rev (5’-AAA GTG TAG CAT ACA ACC CGG CCG TTA CCG GCT ATA TCG AGA TGA GCG AA*G GCT AGT TAA CTA GCC* GAT GAT TAT GAA GAC GGA GGC AAC CAA TTA ACC AAT TCT GAT TAG-3’) were used. The sequences of the stop cassette are indicated by italic type. Insertion of the stop cassette was controlled by restriction enzyme pattern analysis and sequencing. Recombinant CMVs were reconstituted by transfection of purified BAC DNA into MEF using Superfect transfection reagent (Qiagen). Transfected cells were propagated until viral plaques appeared and supernatants from these cultures were used for further propagation. Virus stocks were prepared from supernatants of infected NIH3T3 cells, or from NIH-gO cells in case of gO-transcomplementation, by sucrose-gradient ultracentrifugation as described [41]. Virus titers were determined by TCID_50_ assay or standard plaque assay performed on MEF.

### Quantitation of viral genomes and infectious virus in host tissues

The *in vivo* replication of WT and mutant mCMV was determined by establishing log-linear virus growth curves for various host tissues of interest. At defined times post-infection, viral genomes present in the respective organ lysates were quantitated by M55 (encoding gB)- specific qPCR normalized to cell number by *pthrp* specific qPCR [41]. *In vivo* infectivity was determined from homogenates of infected organs by plaque assay on MEF under conditions of centrifugal enhancement of infectivity.

### *In situ* hybridization specific for viral genes

To distinguish between WT and mutant virus genomes in liver tissue sections, 2-color *in situ* hybridization (2C-ISH) was applied essentially as described previously [41] with hybridization probes adapted to detect or exclude m74 sequences. Probe m74.1 was synthesized using Fluorescein-12-dUTP (Roche Applied Science) in dNTP mix and primer pair m74.1_probe_rev (104.541-CAG AGA CGG TAC GTG TTG-104.558) (GenBank accession no. NC_004065) and m74.1_probe_for (105.150-CGT GTT GGT GAC CGA ATC-105.133). For probe m74.2, Digoxigenin-11-dUTP (Roche Applied Science) was incorporated by PCR using primer pair m74.2_probe_rev (105.280-CCA TGG ATC GGT GAC ACG AAA G-105.301) and m74.2_probe_for (105.774-ATC CGC CGC GAA AGT GAA C-105.746). After DNA hybridization on deparaffinized serial 1-μm sections of liver tissue, red staining was achieved by using alkaline phosphatase-conjugated anti-Fluorescein antibody (Roche Applied Science) with Fuchsin+ Substrate-Chromogen System (Dako) as the chromogenic substrate. Black staining was achieved by using peroxidase-conjugated anti-digoxigenin antibody (Roche Applied Science) with diaminobenzidine tetrahydrochloride (DAB, Sigma-Aldrich) as the substrate, followed by color enhancement with ammonium nickel sulfate hexahydrate.

To detect viral genomes (vDNA) in cell nuclei of infected liver cells for quantitating cells in the late (L) phase of the viral productive cycle, ISH specific for gene M55 was performed on 2-μm sections of liver tissue as described previously [61].

### Immunohistochemical (IHC) analyses of viral protein expression

To simultaneously detect intranuclear viral proteins IE1 and E1 for distinguishing mCMV-infected cells in the immediate-early (IE) and early (E) phase of infection, two-color IHC (2C-IHC) specific for the viral proteins IE1 and E1 was performed on 2-μm liver tissue sections essentially as described [41], with modifications. IE1 was labeled with mAb CROMA 101. Black staining was achieved by using the ImmPRESS HRP anti-mouse Ig detection kit (Vector Laboratories) with DAB as substrate and ammonium nickel sulfate hexahydrate for color enhancement. E1 was labeled with mAb CROMA 103 and stained in red with alkaline phosphatase-conjugated polyclonal goat anti-mouse IgG (AbD Serotec) and Fuchsin+ Substrate-Chromogen System. A light blue counterstaining was achieved with hematoxylin. Single-color IHC specific for glycoprotein B (gB) and the late (L) phase protein MCP (major capsid protein) were performed as described [41].

For quantitating infected cells differentiated by cell type, 3C-IHC analysis was performed on 2-μm liver tissue sections combining intranuclear IE1-specific IHC labeling [41] with the detection of cell type-specific markers CD31 for EC and F4/80 (Ly71) for MΦ: (i) Rat mAb anti-CD31 (PECAM-1; clone SZ31; Dianova) followed by biotin-conjugated polyclonal anti-rat Ig antibody (BD Biosciences) and a peroxidase-coupled avidin biotin complex (Vectastain Elite ABC Kit, Vector Laboratories). (ii) DAB with color enhancement by ammonium nickel sulfate hexahydrate used to stain EC in black, followed by trypsin digestion. (iii) Rat mAb anti-F4/80 (clone BM8; acris antibodies), biotin-conjugated polyclonal anti-rat Ig antibody (BD Biosciences), and peroxidase-coupled avidin biotin complex (Vectastain Elite ABC Kit), followed by HRP-Green Solution Set (42 life sciences) for turquoise-green staining of MΦ. (iv) Red staining of intranuclear IE1 protein with Fuchsin+ Substrate-Chromogen System.

### Statistical analyses and calculation of viral doubling times in host tissues

Statistical tests used are specified in the respective figure legends and were performed using GraphPad Prism version 6.04 for Windows, GraphPad Software. Differences are considered statistically significant for P values of <0.05. Viral doubling times (vDT = log2/*a*) and the corresponding 95% confidence intervals were calculated by linear regression analysis from the slopes *a* of log-linear growth curves [41].

## Supporting Information

S1 Fig Attenuated growth of gO-ko mutants. Multistep virus growth curves characterizing the infection of MEF monolayers with WT mCMV or independent gO-ko mutants ΔgO (Δm74) and ΔgO (m74stop), each at an MOI of 0.05. Supernatants were harvested daily and titrated for infectivity (tissue culture infectious dose, TCID_50_).
(TIF)

S2 Fig Altered patterns of cell culture spread of gO-ko mutants. MEF were infected with WT mCMV or independent gO-ko mutants ΔgO (Δm74) and ΔgO (m74stop). One hour after infection, cell monolayers were washed and incubated for further 3 days under the following conditions: (A) Culture medium alone, (B) culture medium with methylcellulose overlay, (C) culture medium containing neutralizing anti-gB antibodies, and (D) culture medium containing non-neutralizing anti-gB antibodies. Images show foci of infection visualized by indirect immunofluorescent staining of mCMV gB protein.
(TIF)

S3 Fig Reversal of the ΔgO virus growth deficiency in organs of immunocompromised adult mice by gO-transcomplementation. Adult BALB/c mice were immunocompromised (5.5 Gy of γ-irradiation) and infected i.v. with 10^3^ PFU of the indicated viruses. Viral infectivity in organ homogenates (PFU/organ for spleen and lungs; PFU/g for the liver) was quantitated on day 8 by virus plaque assay. Symbols represent data from individual mice with the median values marked. DL, detection limit. For statistical analysis of differences between experimental groups, log-normal distribution was verified using the distribution-free Kolmogorov-Smirnov test (D statistics). P values were calculated from log-transformed data using Student’s t-test (unpaired, two-sided) with Welch’s correction to account for unequal variance.
(TIF)

S4 Fig Verification of the genetic authenticity of virus ΔgO-gO^trans^. To rule out genetic recombination might have occurred unintendedly during propagation of virus ΔgO-gO^trans^ with vector sequence in the gO-transcomplementing transfectant cell line NIH-gO, absence of gO DNA sequence was verified by 2C-ISH in liver tissue sections of immunocompromised BALB/c mice (6.5 Gy of γ-irradiation) on day 10 after i.v. infection with 1x10^3^ PFU each of either WT virus or ΔgO-gO^trans^ virus or both upon coinfection. (A) Differential *in situ* hybridization strategy for distinguishing between viruses carrying or lacking gO-encoding m74 sequence. Shown is a genome map (not drawn to scale) with positions of probe m74.1 (red stain), specific for sequence shared between WT and mutant, and of probe m74.2 (black stain) specific for sequence deleted in the mutant. Nucleotide positions refer to the 5’ end of ORF m74. (B) Chessboard scheme of 2C-ISH images with viruses and hybridization probes indicated. For each type of infection (columns), three consecutive 1-μm tissue sections (see landmarks) were taken to hybridize viral DNA from precisely the same infection foci. Bar marker: 100 μm.
(TIF)

S5 Fig Comparison of relative infection efficiencies of ΔgO mutants and gO-transcomplemented ΔgO mutant ΔgO-gO^trans^ for different cell types in culture. Diluted virus stocks of the indicated viruses were used to infect adherent cells. Proportions of infected cells (for all viruses normalized to the number of infected primary fibroblasts (MEF), which were infected in parallel with virus doses resulting in infections of 20% to 50% of the cells), were determined at (A) 4h p.i. by indirect immunofluorescence or (B) 16 h p.i. by intracellular cytofluorometric analysis specific for the IE1 protein. Cell types analyzed are represented by cell lines NIH3T3 (fibroblasts), TCMK-1 (epithelial cells), MHEC-5T (EC), and ANA-1 (MΦ). Bars represent means +/- SD of at least three independent experiments.
(TIF)

S6 Fig Proportions of infected liver cells classified by cell type. Data refer to the experiment shown in Fig. 3 for WT virus. Infected and uninfected cells of the indicated 3 cell types were identified by 3C-IHC at 24h after infection. Cell numbers given on the ordinate refer to representative 10-mm^2^ areas of liver tissue sections. Bars indicate median values of data from 3 individual mice analyzed. Variance bars indicate the range. P values for the significance of differences in the percentages of infected cells were calculated by using the ratio paired t-test.
(TIF)

S7 Fig The alternative gH/gL complex gH/gL/MCK-2 is not essential for virus entry and spread in the liver. Data come from the experiment shown in Fig. 8B and reveal congruency in the time course of the viral DNA load in the liver after infection by viruses WT (filled circles) and ΔMCK-2 (open circles). Symbols in the two single virus panels represent data from individual mice, symbols in the merge (outer right) panel represent the corresponding median values. For the explanation of log-linear regression analysis (calculating vDT), see the legend of Fig. 4.
(TIF)

S8 Fig gO-independent virus spread in fibroblast cell culture is inhibited by genetic MCK-2-ko or by blocking antibody. MEF monolayers were infected with viruses ΔgO-gO^trans^ (outer left and outer right images) or ΔgOΔMCK-2-gO^trans^ (center images). One hour after infection, cell monolayers were washed and incubated for further 3 days with culture medium containing a control rabbit antiserum (outer left images), culture medium containing rabbit anti-MCK-2 serum (outer right images), or just culture medium (center images). Photographs show foci of infection visualized by indirect immunofluorescent staining for mCMV gB (upper panel) or intranuclear IE1 protein (lower panel).
(TIF)
