# Supplementary figures and images for: Non-redundant and Redundant Roles of Cytomegalovirus gH/gL Complexes in Host Organ Entry and Intra-tissue Spread
Source: PLoS Pathog. 2015 Feb 6;11(2):e1004640. doi: 10.1371/journal.ppat.1004640 (PMC4450070; doi:10.1371/journal.ppat.1004640)

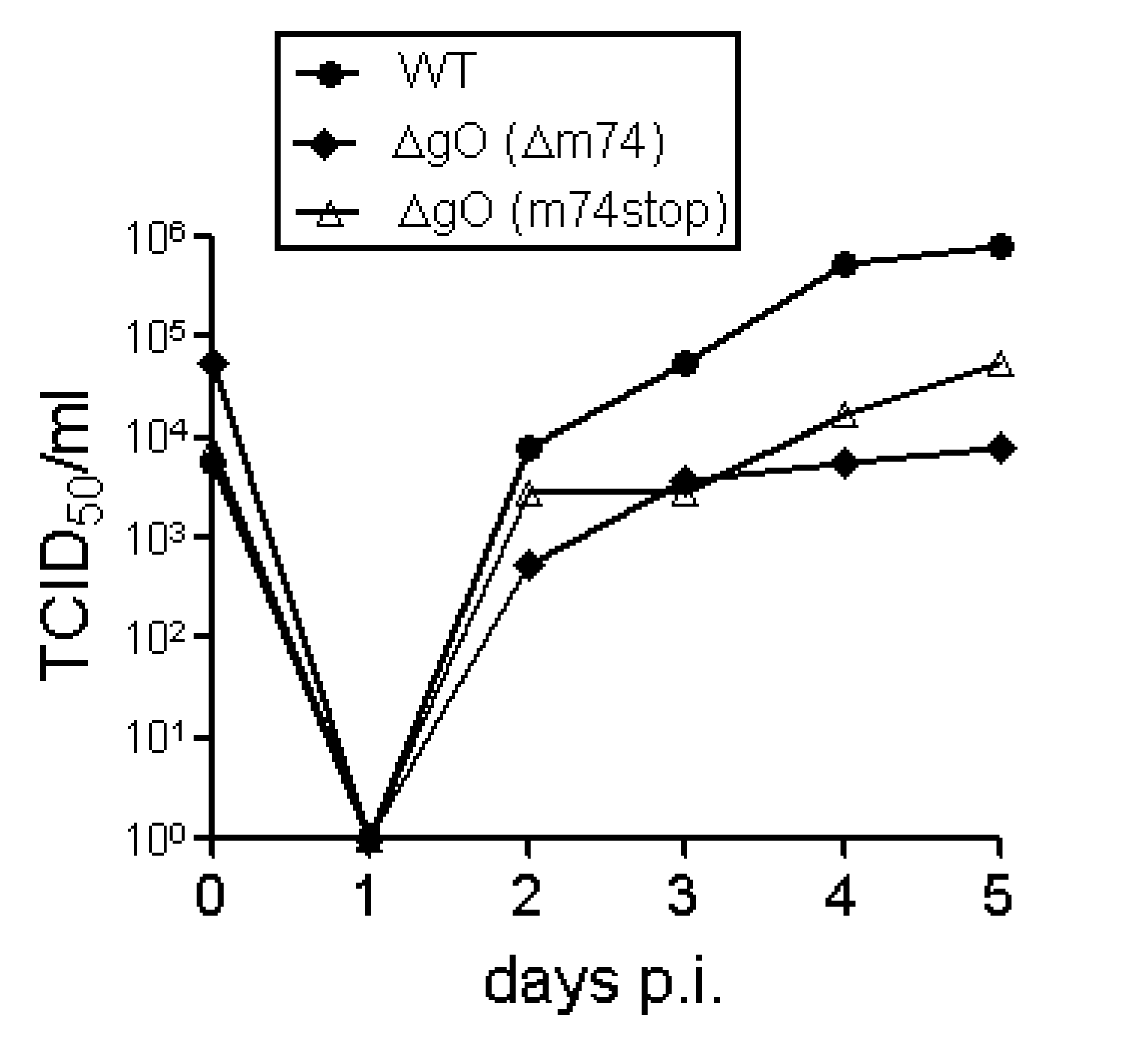

Supplement: S1 Fig — Multistep virus growth curves characterizing the infection of MEF monolayers with WT mCMV or independent gO-ko mutants ΔgO (Δm74) and ΔgO (m74stop), each at an MOI of 0.05. Supernatants were harvested daily and titrated for infectivity (tissue culture infectious dose, TCID50). (TIF) [file ppat.1004640.s001.tif]

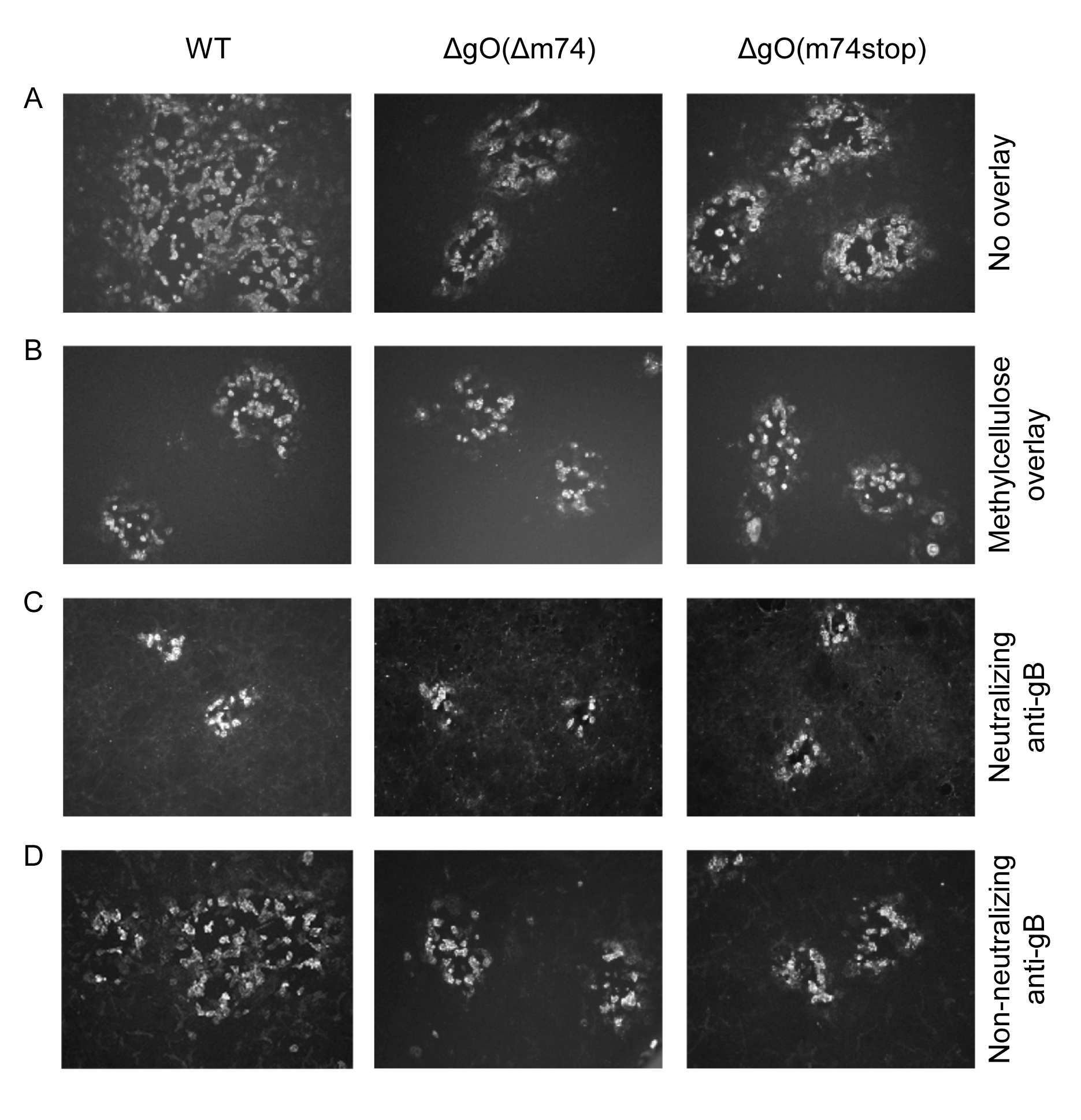

Supplement: S2 Fig — MEF were infected with WT mCMV or independent gO-ko mutants ΔgO (Δm74) and ΔgO (m74stop). One hour after infection, cell monolayers were washed and incubated for further 3 days under the following conditions: (A) Culture medium alone, (B) culture medium with methylcellulose overlay, (C) culture medium containing neutralizing anti-gB antibodies, and (D) culture medium containing non-neutralizing anti-gB antibodies. Images show foci of infection visualized by indirect immunofluorescent staining of mCMV gB protein. (TIF) [file ppat.1004640.s002.tif]

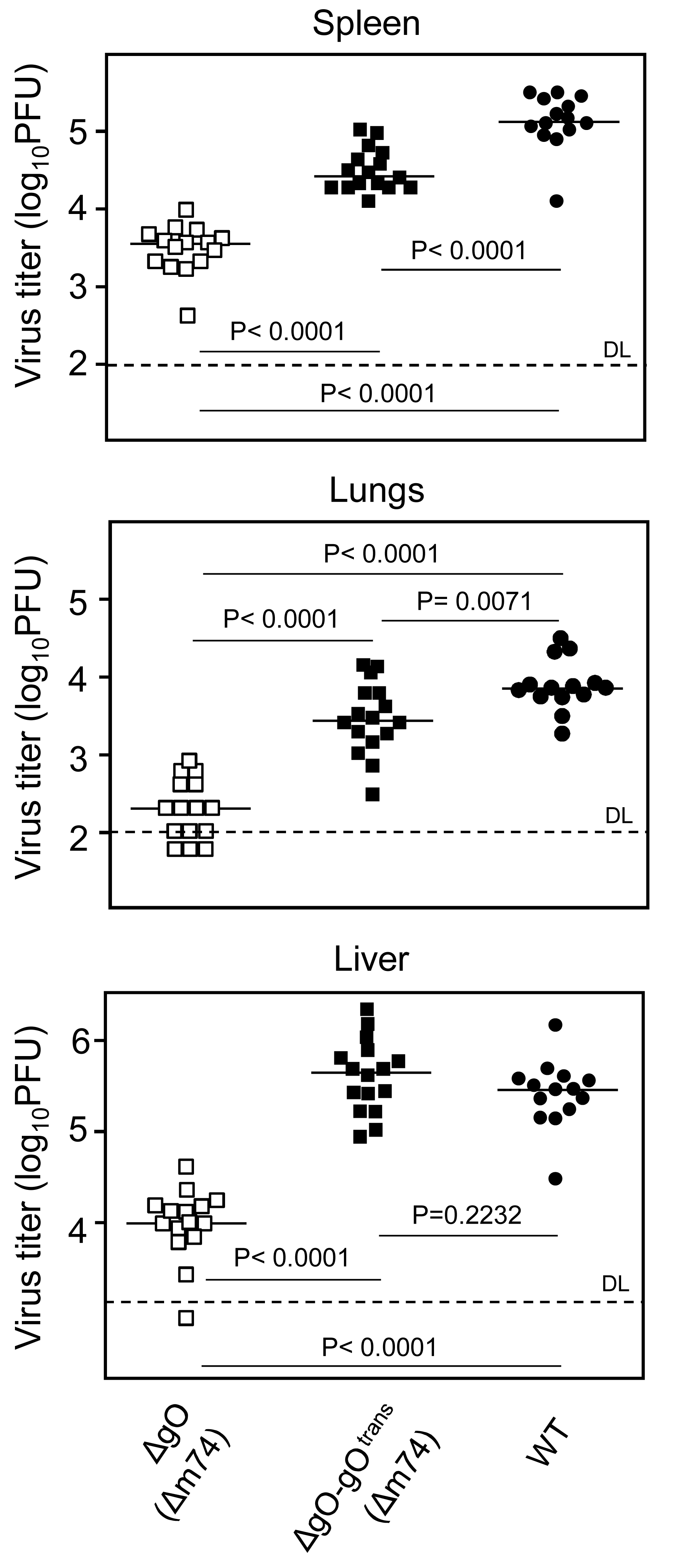

Supplement: S3 Fig — Adult BALB/c mice were immunocompromised (5.5 Gy of γ-irradiation) and infected i.v. with 103 PFU of the indicated viruses. Viral infectivity in organ homogenates (PFU/organ for spleen and lungs; PFU/g for the liver) was quantitated on day 8 by virus plaque assay. Symbols represent data from individual mice with the median values marked. DL, detection limit. For statistical analysis of differences between experimental groups, log-normal distribution was verified using the distribution-free Kolmogorov-Smirnov test (D statistics). P values were calculated from log-transformed data using Student’s t-test (unpaired, two-sided) with Welch’s correction to account for unequal variance. (TIF) [file ppat.1004640.s003.tif]

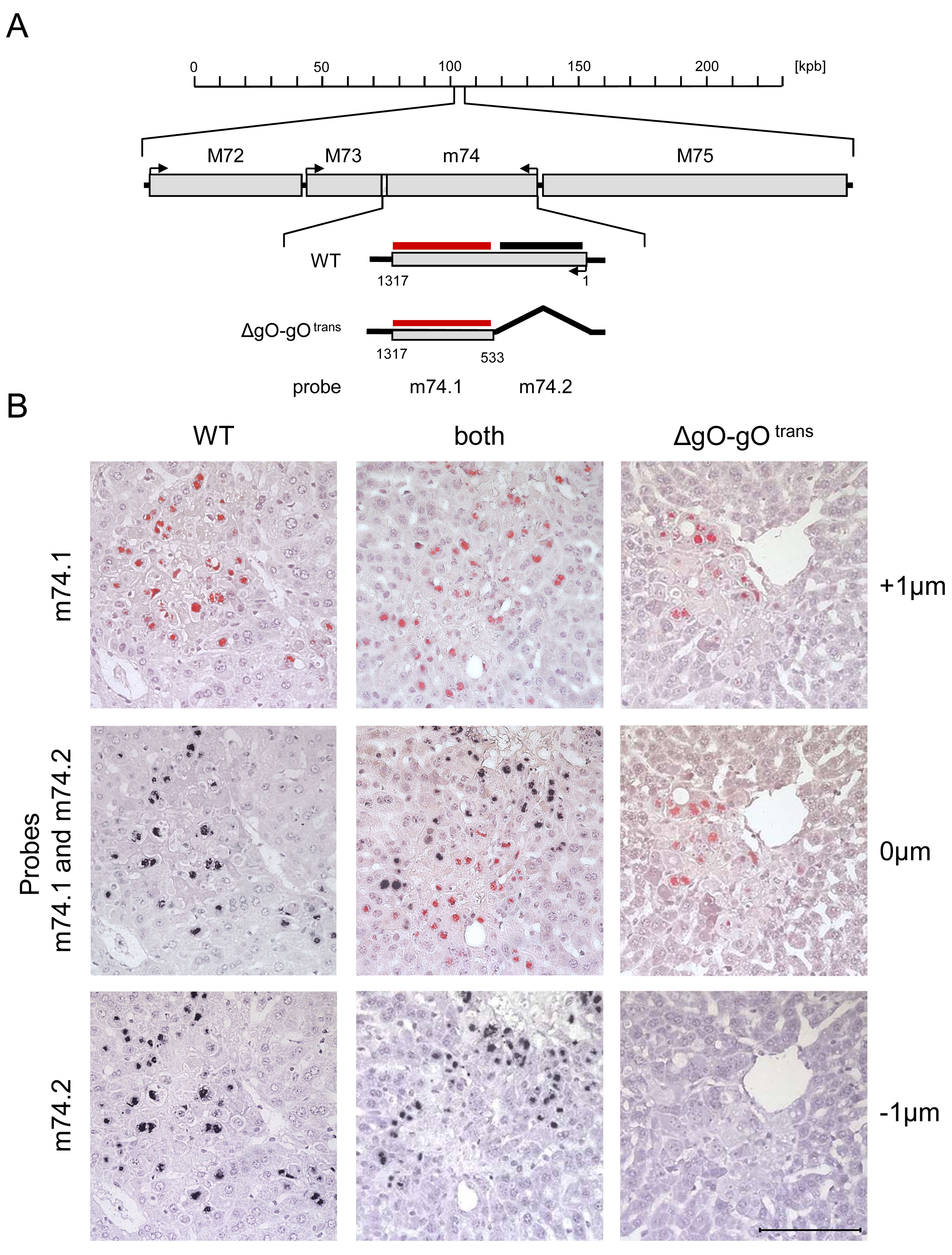

Supplement: S4 Fig — To rule out genetic recombination might have occurred unintendedly during propagation of virus ΔgO-gOtrans with vector sequence in the gO-transcomplementing transfectant cell line NIH-gO, absence of gO DNA sequence was verified by 2C-ISH in liver tissue sections of immunocompromised BALB/c mice (6.5 Gy of γ-irradiation) on day 10 after i.v. infection with 1x10^3^ PFU each of either WT virus or ΔgO-gOtrans virus or both upon coinfection. (A) Differential in situ hybridization strategy for distinguishing between viruses carrying or lacking gO-encoding m74 sequence. Shown is a genome map (not drawn to scale) with positions of probe m74.1 (red stain), specific for sequence shared between WT and mutant, and of probe m74.2 (black stain) specific for sequence deleted in the mutant. Nucleotide positions refer to the 5’ end of ORF m74. (B) Chessboard scheme of 2C-ISH images with viruses and hybridization probes indicated. For each type of infection (columns), three consecutive 1-μm tissue sections (see landmarks) were taken to hybridize viral DNA from precisely the same infection foci. Bar marker: 100 μm. (TIF) [file ppat.1004640.s004.tif]

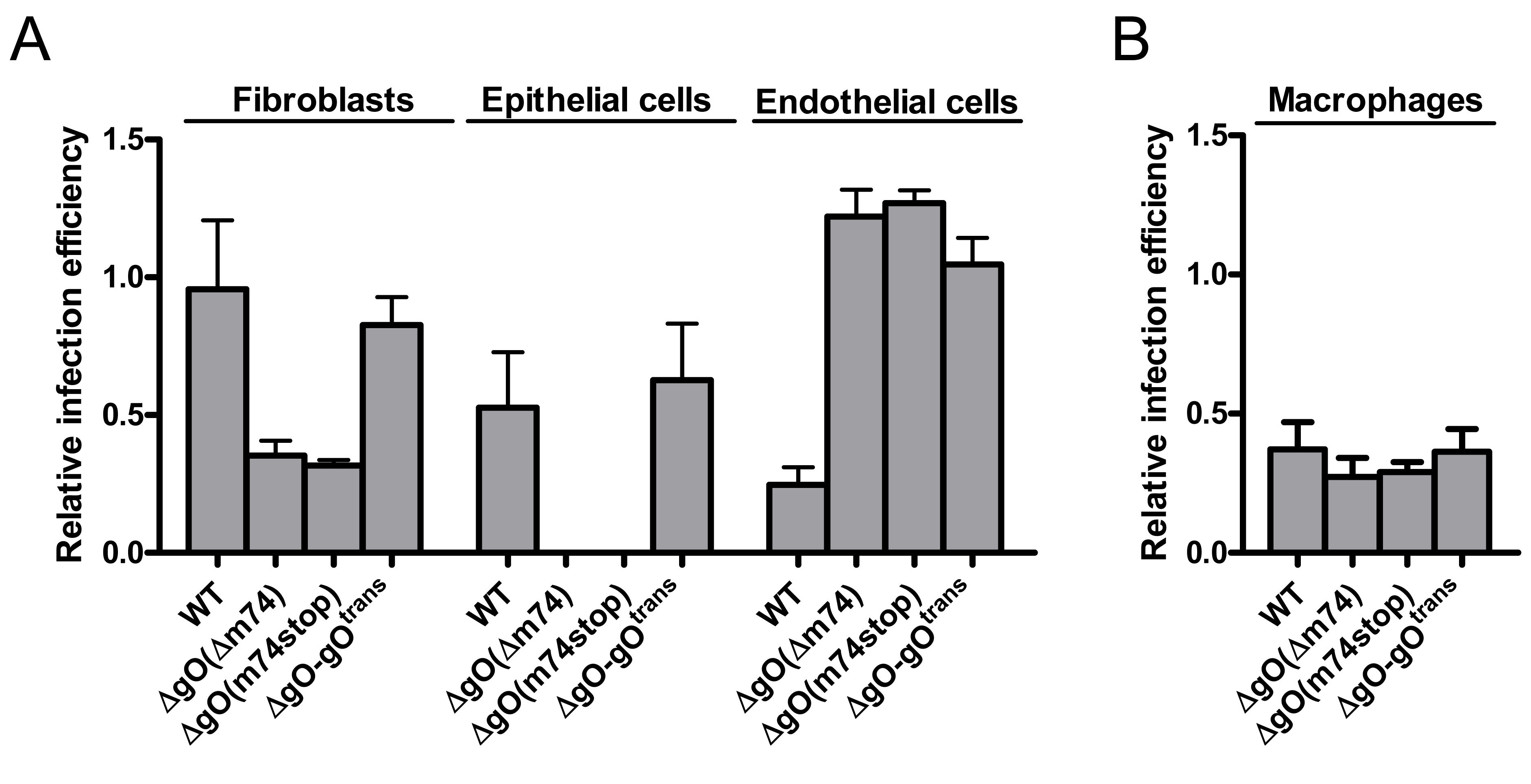

Supplement: S5 Fig — Diluted virus stocks of the indicated viruses were used to infect adherent cells. Proportions of infected cells (for all viruses normalized to the number of infected primary fibroblasts (MEF), which were infected in parallel with virus doses resulting in infections of 20% to 50% of the cells), were determined at (A) 4h p.i. by indirect immunofluorescence or (B) 16 h p.i. by intracellular cytofluorometric analysis specific for the IE1 protein. Cell types analyzed are represented by cell lines NIH3T3 (fibroblasts), TCMK-1 (epithelial cells), MHEC-5T (EC), and ANA-1 (MΦ). Bars represent means +/- SD of at least three independent experiments. (TIF) [file ppat.1004640.s005.tif]

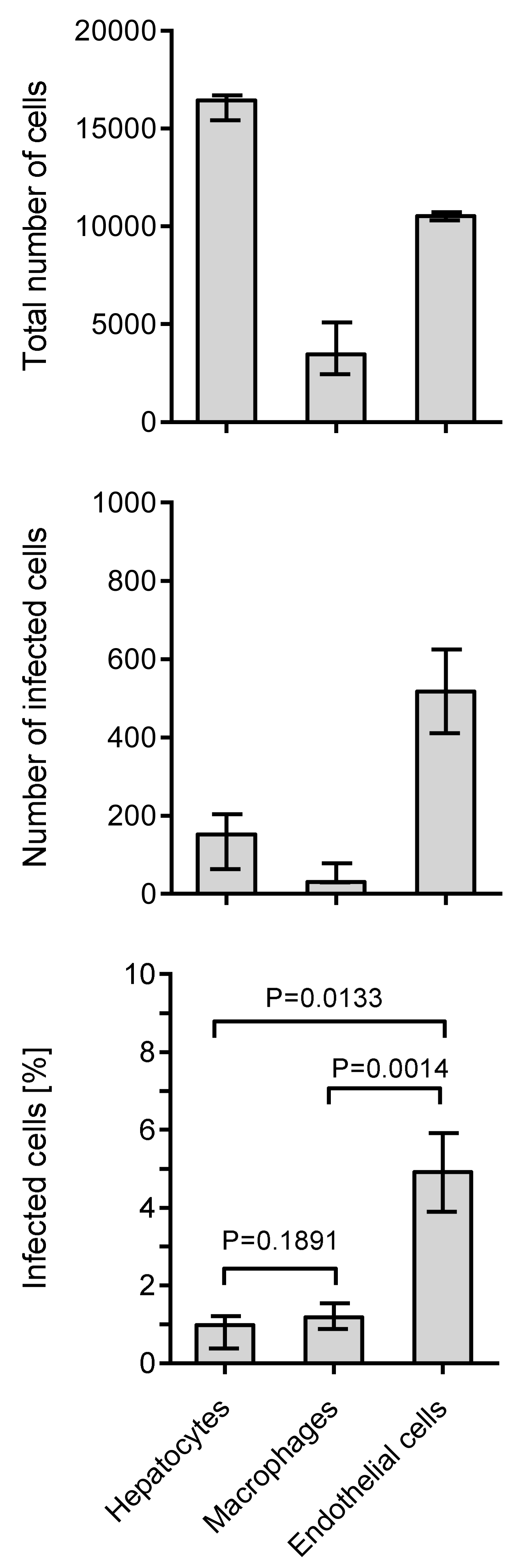

Supplement: S6 Fig — Data refer to the experiment shown in Fig. 3 for WT virus. Infected and uninfected cells of the indicated 3 cell types were identified by 3C-IHC at 24h after infection. Cell numbers given on the ordinate refer to representative 10-mm2 areas of liver tissue sections. Bars indicate median values of data from 3 individual mice analyzed. Variance bars indicate the range. P values for the significance of differences in the percentages of infected cells were calculated by using the ratio paired t-test. (TIF) [file ppat.1004640.s006.tif]

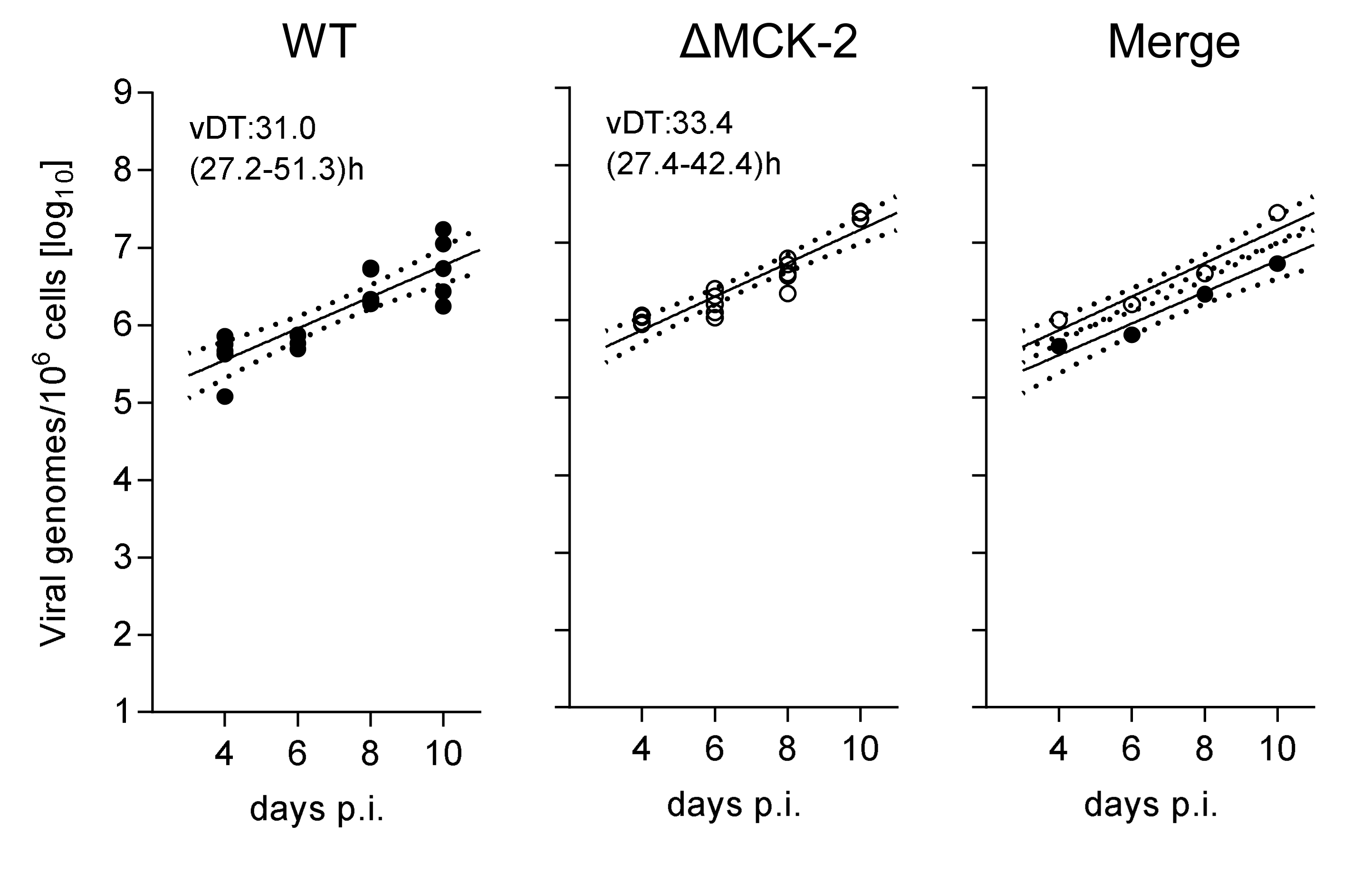

Supplement: S7 Fig — Data come from the experiment shown in Fig. 8B and reveal congruency in the time course of the viral DNA load in the liver after infection by viruses WT (filled circles) and ΔMCK-2 (open circles). Symbols in the two single virus panels represent data from individual mice, symbols in the merge (outer right) panel represent the corresponding median values. For the explanation of log-linear regression analysis (calculating vDT), see the legend of Fig. 4. (TIF) [file ppat.1004640.s007.tif]

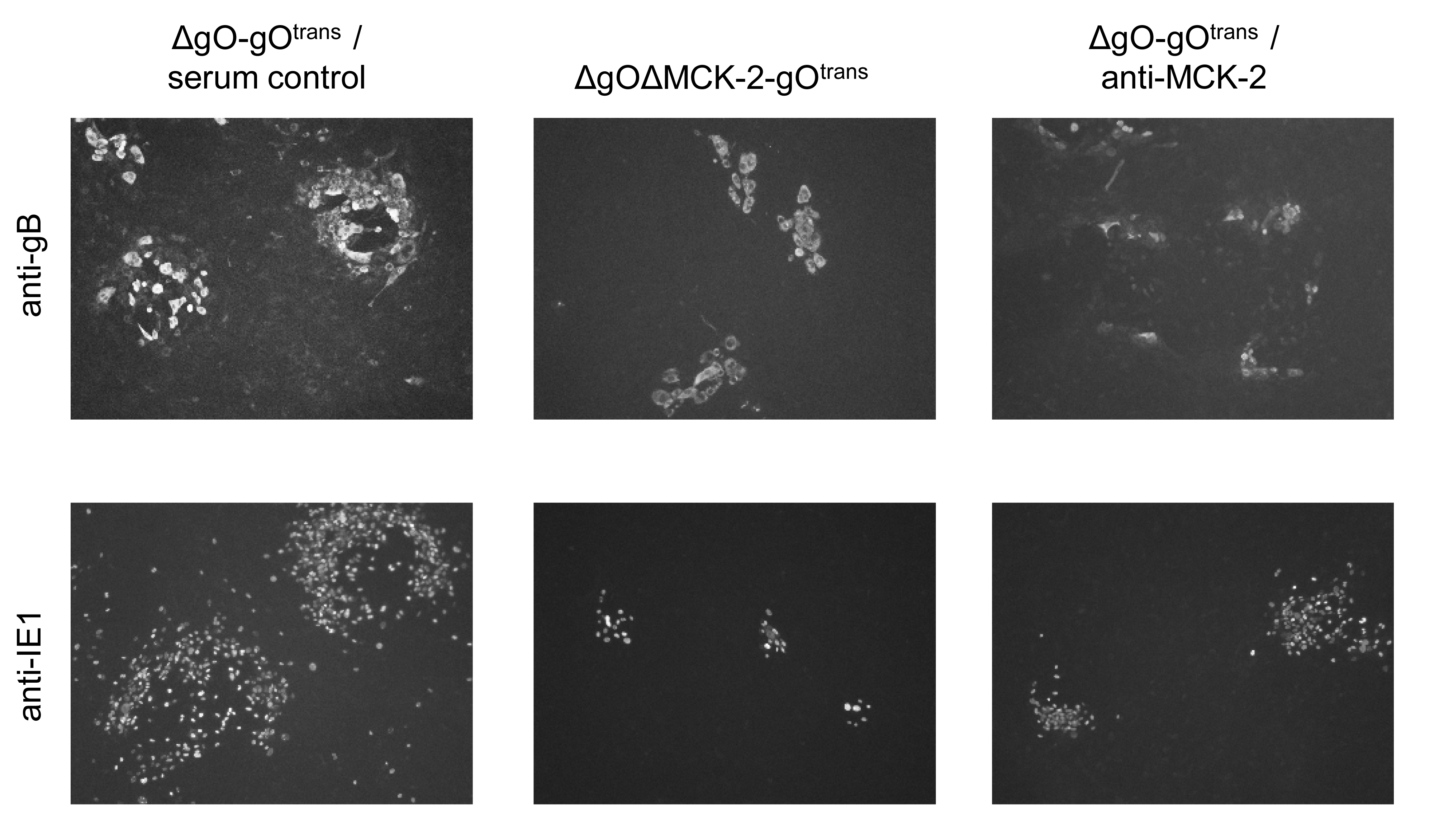

Supplement: S8 Fig — MEF monolayers were infected with viruses ΔgO-gOtrans (outer left and outer right images) or ΔgOΔMCK-2-gOtrans (center images). One hour after infection, cell monolayers were washed and incubated for further 3 days with culture medium containing a control rabbit antiserum (outer left images), culture medium containing rabbit anti-MCK-2 serum (outer right images), or just culture medium (center images). Photographs show foci of infection visualized by indirect immunofluorescent staining for mCMV gB (upper panel) or intranuclear IE1 protein (lower panel). (TIF) [file ppat.1004640.s008.tif]
